# Dysregulated IGFBP5 expression causes axon degeneration and motoneuron loss in diabetic neuropathy

**DOI:** 10.1007/s00401-015-1446-8

**Published:** 2015-05-30

**Authors:** Christian M. Simon, Stefanie Rauskolb, Jennifer M. Gunnersen, Bettina Holtmann, Carsten Drepper, Benjamin Dombert, Massimiliano Braga, Stefan Wiese, Sibylle Jablonka, Dirk Pühringer, Jürgen Zielasek, Andreas Hoeflich, Vincenzo Silani, Eckhard Wolf, Susanne Kneitz, Claudia Sommer, Klaus V. Toyka, Michael Sendtner

**Affiliations:** Institute for Clinical Neurobiology, University of Würzburg, Versbacher-Str. 5, 97078 Würzburg, Germany; Department of Neurology, University of Würzburg, Josef-Schneider-Str. 11, 97080 Würzburg, Germany; A.O. Desio e Vimercate, Via Santi Cosma e Damiano 10, 20871 Vimercate, Italy; Department of Pathophysiology and Transplantation, “Dino Ferrari” Center, Università degli Studi di Milano, Via Francesco Sforza 35, 20122 Milan, Italy; Institute of Molecular Animal Breeding and Biotechnology, Gene Center, LMU Munich, Feodor-Lynen Str. 25, 81377 Munich, Germany; Department of Physiological Chemistry I, Biocenter, University of Würzburg, Am Hubland, 97074 Würzburg, Germany; Motor Neuron Center, Columbia University, 630 West 168th Street, Room 4-401, New York, NY 10032 USA; Department of Anatomy and Neuroscience, The University of Melbourne, Parkville, VIC 3010 Australia; Central Facility for Biological and Biomedical Research, University of Hohenheim, Schwerzstrasse 15/1, 70593 Stuttgart, Germany; Department of Child and Adolescent Psychiatry, Psychosomatics, and Psychotherapy, University Hospital of Würzburg, Füchsleinstr.15, 97080 Würzburg, Germany; Group of Cell Morphology and Molecular Neurobiology, Building NDEF 05/598, Universitätsstr. 150, Ruhr-University Bochum, 44801 Bochum, Germany; Department for Obstetrics and Gynecology, University of Würzburg, Josef-Schneider-Str. 4, 97080 Würzburg, Germany; Medical Faculties of the Heinrich-Heine University, Bergische Landstraße 2, 40629 Düsseldorf, Germany; Institute of Genome Biology, Leibniz Institute for Farm Animal Biology (FBN), Wilhelm-Stahl-Allee 2, 18196 Dummerstorf, Germany; Department of Neurology and Laboratory of Neuroscience, IRCCS Instituto Auxologico Italiano, Piazzale Brescia 20, 20149 Milan, Italy

**Keywords:** Motor nerve biopsy, Diabetic polyneuropathy, Neuropathy, Neurotrophic factors, Axonal degeneration

## Abstract

Diabetic neuropathy (DNP), afflicting sensory and motor nerve fibers, is a major complication in diabetes. The underlying cellular mechanisms of axon degeneration are poorly understood. IGFBP5, an inhibitory binding protein for insulin-like growth factor 1 (IGF1) is highly up-regulated in nerve biopsies of patients with DNP. We investigated the pathogenic relevance of this finding in transgenic mice overexpressing IGFBP5 in motor axons and sensory nerve fibers. These mice develop motor axonopathy and sensory deficits similar to those seen in DNP. Motor axon degeneration was also observed in mice in which the IGF1 receptor (IGF1R) was conditionally depleted in motoneurons, indicating that reduced activity of IGF1 on IGF1R in motoneurons is responsible for the observed effect. These data provide evidence that elevated expression of IGFBP5 in diabetic nerves reduces the availability of IGF1 for IGF1R on motor axons, thus leading to progressive neurodegeneration. Inhibition of IGFBP5 could thus offer novel treatment strategies for DNP.

**Electronic supplementary material:**

The online version of this article (doi:10.1007/s00401-015-1446-8) contains supplementary material, which is available to authorized users.

## Introduction

Diabetic neuropathy (DNP) is a heterogeneous disorder, with sensory, motor, and autonomic system involvement [5], including small fiber disease, all leading to progressive disability [37, 40]. The etiopathogenesis of DNP is complex and involves metabolic, vascular, and immune-mediated components [50], but whether trophic factor dysregulation plays a role is still unclear. In a large study comparing disability in the elderly, motor performance was more reduced in diabetic than non-diabetic individuals and found to make a major contribution to overall disability [45]. In streptozotocin-treated mice, a widely used rodent model of some aspects of DNP, progressive loss of motor fibers becomes apparent with age, with loss of distal axons occurring prior to proximal axons and motoneuron cell bodies [39]. In the same model, reduced insulin-like growth factor 1 (IGF1) expression [53] and elevated renal insulin-like growth factor-binding protein 5 (IGFBP5) levels were found [35], but the relevance of these findings for DNP remained unclear. IGF1 and IGF2 are pluripotent growth and survival factors for a variety of cell types [42]. They support the survival of motoneurons in vitro and in vivo [20, 33]. *Igf1* knockout mice show multiple defects including reduced myelination and loss of striatal neurons [3]. IGF1 is expressed in Schwann cells of peripheral nerves in developing and postnatal rodents [10]. It acts on Schwann cells, in particular by stimulating myelination [11, 46], and possibly also on myelinated axons [31]. In transgenic mice overexpressing IGF1, enhanced myelin production was among the most prominent effects [9, 55].

The biological functions of IGF1 and IGF2 are modulated by at least 6 binding proteins (IGFBPs) [14, 27, 54]. Some of these binding proteins are produced in the liver and function as carriers for IGFs in the circulation [13]. Other IGFBPs are expressed in specific tissues and are thought to increase local concentrations of IGFs by trapping circulating IGF1 and/or IGF2, as well as to compete with cellular receptors for IGF binding [1, 24]. Thus, they are potential regulators of the local availability and biological functions of IGFs. IGFBP5, a 29-kDa glycosylated protein [15], is a high affinity binding protein for IGF1, which, at least in vitro, inhibits receptor binding of IGFs resulting in reduced receptor autophosphorylation [26]. IGFBP5 is widely expressed in the body including in the kidney [35, 38] and the nervous system [4, 44]. In postnatal peripheral nerves, IGFBP5 immunoreactivity is detectable in close association with or within myelinated axons, suggesting that it is anterogradely transported and released from axons [12, 46]. IGFBP5 binds with high affinity to extracellular matrix (ECM) [14, 25]. Thus, IGFBP5 may be involved in regulating local functions of IGF1 and IGF2 at the interface between motoneurons and Schwann cells. To study the role of the IGF1/IGFBP5/IGF1R system for axon maintenance, we investigated alterations of expression of IGFs and IGFBPs in peripheral nerves of patients with DNP and compared them with other neuropathies and healthy controls. We found that IGFBP5 levels are increased in sural nerve biopsies of patients with sensorimotor DNP. To determine the pathogenic role of elevated IGFBP5, we established transgenic mouse lines in which IGFBP5 is overexpressed specifically in neurons under the control of the neurofilament-light chain (NF-L) promoter and found a progressive neuropathy in these mutants that affected both sensory and motor nerve fibers. We also tested whether reduced availability of IGF1 may be responsible for the degeneration of motoneurons by establishing mice in which the IGF1R is inactivated in motoneurons. In these mice, we observed a progressive degeneration of motor axons and cell bodies. These results indicate that elevated expression of IGFBP5 along with inhibition of IGF1 signaling in motoneurons leads to degeneration of their axons and cell bodies and suggest a causative link. Thus, increased expression of IGFBP5 may play a role in the pathogenesis of sensory defects and motor axonopathy associated with DNP.

## Materials and methods

### Animals

The generation of the *Igfbp5 tg*+ (*Bp5tg*+) and *cIgf1r ko* mice is described in the supplementary methods section. Analyses with these mouse lines were performed between the 4th and the 12th generation of backcrossing of these mice onto a C57Bl/6J background. Animals were kept in a temperature-controlled environment with a 12-h-light/12-h-dark cycle. For details of behavioral analyses see supplementary methods section.

### Sural nerve biopsies

Diagnostic nerve biopsies were obtained from the Department of Neurology, Würzburg. Diabetic neuropathy (DNP, *n* = 6, one type I, five type II) was diagnosed according to established criteria [34] after excluding other causes of polyneuropathy [49]. Chronic inflammatory demyelinating polyradiculoneuropathy (CIDP, *n* = 9) was diagnosed according to INCAT criteria [19]. DNP in combination with CIDP [23] was diagnosed in four patients with type II diabetes showing marked motor impairment and prominent demyelination in the sural nerve with reduced nerve conduction velocities thus fulfilling the INCAT criteria [19] for CIDP. All 19 patients had motor nerve abnormalities when tested by standard nerve conduction testing and electromyography [21, 28]. Samples from three patients with non-diabetic neuropathy were included as disease controls: mild axonal neuropathy with motor neuron disease (*n* = 2) and vitamin B12 deficiency (*n* = 1). Non-diseased control tissues (*n* = 5) were taken from biopsy and autopsy material that was checked for absence of pathology. For details see electronic supplementary Table A2.

### Immunohistochemistry

Human nerves were fixed for 24 h in 4 % PFA at 4 °C. 10 μm frozen transverse nerve sections were prepared. Mice were perfused with 4 % PFA and sciatic nerves were dissected. Sections were incubated overnight with primary antibodies: neurofilament (1:500, Covance), IGFBP5 (1:200, Abcam), laminin B1 (1:500; Millipore). Sections were then washed and incubated with secondary antibodies conjugated to FITC (1:40, Dako), Alexa Fluor 633 (1:500, Invitrogen) or Cy3 (1:600, Jackson ImmunoResearch Laboratories, Inc) for 1 h, mounted and investigated with the Olympus FluoView™ FV1000 microscope.

### Motoneuron culture

Murine embryonic spinal motoneurons were cultured as described [52]. BDNF, human IGF1 (PeProTech) and mouse IGFBP5 (BP5DU020, GroPep) were used at the concentrations indicated. Cells were initially counted 4 h after plating to obtain the reference value for 100 % survival. After 7 DIV, surviving motoneuron cells were counted again. For IGF1 stimulation experiments, motoneurons were cultured for 5 days with 5 ng/ml BDNF in full medium, and then starved in the same medium without horse serum and BDNF for 12 h. Cells were then stimulated with IGF1. Neurons were fixed with 4 % PFA and stained with anti-Tau (1:1000, Sigma) and anti-IGFBP5 (1:500, Neuromics) antibodies. Cellular morphology was visualized with fluorescence-coupled secondary antibodies (Jackson ImmunoResearch) using a Leica SP2 confocal microscope. Measurements were done with the Leica AS Lite software (Leica).

### Dorsal root ganglionic (DRG) sensory neuronal culture

Murine embryonic DRGs were isolated and cultured as described previously [22]. For IGF1 stimulation experiments, DRG neurons were cultured with neurobasal medium (NB) in the presence of 2 % horse serum, 2 % B27 and 10 ng/ml NGF (PeProTech) for 20 h. Cells were starved for 4 h in NB after three washing steps with NB to minimize NGF levels. DRGs were then stimulated with either 10 ng/ml NGF, 20 ng/ml human IGF1 (PeProTech) or 20 ng/ml human IGF1 and 200 ng/ml mouse IGFBP5 (BP5DU020, GroPep), respectively. Subsequently, cells were washed with phosphate buffer saline and lysed for 10 min at 4 °C with lysis buffer comprising 50 mM Tris–HCl (pH 7.4), 150 mM NaCl, 1 % Triton-X-100, Protease- and Phosphatase inhibitors (Thermo Scientific). Equal volumes of DRG cell lysates were loaded onto 12 % SDS gels and immunoblotted for IGF1R, pIGF1R, AKT, pAKT and calnexin.

### Western blot analysis

Protein was isolated from nerve biopsies homogenized in lysis buffer (150 mM NaCl, 1 % Triton, 2 mM EDTA, 50 mM Tris, pH 7.4). 30 µg protein extract was electrophoresed on a 12 % SDS-PAGE gel and blotted for 40 min to PVDF membrane. The membranes were probed with rabbit anti-IGFBP5 (H-100, 1:5000, Santa Cruz Biotechnology) for human samples and goat anti-IGFBP5 antibody (GT15183, 1:5000, Neuromics) for murine samples in 5 % skim milk, and rabbit anti-phosho-IGF1 receptor beta (3918, 1:2000) in 5 % BSA, anti-IGF1 receptor beta (3027, 1:2000) in 5 % milk, for immunoprecipitation 1:1000 in combination with protein A agarose beads (11719386001, Roche), anti-phospho-Akt (9271, 1:2000 in 5 % BSA) and anti-Akt (9272, 1:2000 in 5 % milk, all from Cell Signaling Technology) for mouse extracts, in blocking buffer for 1 h. The appropriate HRP-conjugated secondary antibody (Jackson ImmunoResearch Laboratories, Inc) was used and visualized using enhanced chemiluminescence (GE Healthcare, Lifesciences). The blots were reprobed with mouse anti-actin antibody (Clone C4, 1:7000, Millipore). Film images were scanned and the intensity of IGFBP5 was standardized to mouse anti-actin. A minimum of *n* = 3 per group were tested in 3 independent experiments.

### Quantitative morphometry on cross sections of the phrenic and sciatic nerve

The morphological analysis of phrenic and sciatic nerve axons was carried out in *Igfbp5* transgenic mice derived from mouse line 8 at 3 weeks (wt: *n* = 4; *Bp5 tg*+: *n* = 6) and 5 to 6 months (wt: *n* = 5; *Bp5 tg*+: *n* = 3) of age and in 5- to 6-month-old transgenic mice derived from mouse line 9 (*n* = 3). In addition, the phrenic and sciatic nerve axons were analyzed in 4 *cIgf1r ko* and 4 wild-type mice at 6 months of age. Mice were transcardially perfused with 0.1 M phosphate buffer followed by a mixture of 4 % paraformaldehyde and 2 % glutaraldehyde in 0.1 M cacodylate buffer. The proximal part of the phrenic nerves was then dissected and postfixed in the same fixative overnight. After osmification and dehydration, all samples were embedded in Spurr`s medium. Semithin (1 µm) cross sections for light microscopic examination were cut with a diamond knife on an ultramicrotome. Sections were stained with azur–methylene blue for histomorphological analysis and subsequent morphometric evaluation. The nerve sections of *Igfbp5 tg*+ mice were analyzed under a light microscope (Leica GmbH, Bensheim, Germany) and morphometric evaluation of phrenic and sciatic nerves was performed using the Quantimed 500 morphometric system (Leica GmbH, Bensheim, Germany). The number of intact myelinated fibers in motoneuron-specific *Igf1r* mutant mice was determined from photographs taken from nerve cross sections under an Leica (Nussloch, Germany) light microscope equipped with a digital camera (ActionCam; Agfa, Mortsel, Belgium). To estimate the total number and relative axon size distributions in sciatic nerves, 4 *cIgf1r ko* and 4 wild-type mice were prepared as described above. A segment of the main sciatic nerve trunk was taken from a standard site. Cross sections were photographed using a Zeiss Axiophot microscope in conjugation with a digital Spot Insight Camera with corresponding Spot Software (Visitron Systems GmbH, Puchheim, Germany). The stereological analysis of myelinated axons was carried out on a Vario Vision Docu software analysis system (Vario Vision Docu; Soft Imaging System, Münster, Germany). To obtain unbiased number and size estimates from a sample of axons in a nerve cross section, we followed the concept of systematic random sampling using a systematically positioned unbiased counting frame [18]. The entire nerve cross sections were photographed at low magnification (200×) and the entire nerve cross section areas were determined. Approximately 10 high magnification photomicrographs (1000×) were taken from each nerve cross section. The counting frame (2500 µm^2^) was systematically placed into the lower right corner of each photomicrograph covering at least one-half of the nerve cross section. Axons in the frame or at the (dashed) inclusion lines were counted provided that they do not in any way touch the (solid) exclusion lines or their infinite extensions. Approximately 300–600 axons were counted in 6–8 micrographs per nerve cross section. Estimates of the total number of axons were calculated as: total number of axons counted divided by the product of number and area of the counting frames multiplied by the area of the nerve cross section. Absolute and relative size distributions of myelinated axons were measured on the same photomicrograph so that number and size estimations were combined into one complete procedure. The cross-sectional area of the axons was determined by tracing them with a cursor on a digitizing tablet of the Vario Vision Docu analysis system. The M-ratio was calculated by the division of myelin thickness by axon diameter. At least 150 M-ratios and circumferences per nerve were measured in at least 3 individuals per group.

### Intraepidermal nerve fiber density (IENFD)

Foot pads were collected from the plantar surface of the hind paw, immersion fixed with 4 % paraformaldehyde for 2 h, cryoprotected overnight in 30 % sucrose, cryoembedded in mounting media (OCT), and 12 μm cryostat sections were prepared. Sections were labeled using rabbit polyclonal antibodies against PGP9.5 (1:800, Ultra clone limited, RA95101) and donkey anti-rabbit (H+L) IgG Cy3-conjugated secondary antibodies (1:700, Jackson Immunoresearch). Images were acquired using a Leica SP2 confocal microscope system (Leica Microsystems) and a 40× oil immersion objective. Eight confocal images were captured at 1 µm intervals and an average projection image was produced by ImageJ (MacBiophotonics). Images were analyzed using the Leica LAS AF Lite software. Individual intraepidermal nerve fibers which passed through the basement membrane were scored from individual sections of independent animals. In total, 16 sections from footpads from 4 wild-type and 21 sections from footpads from 4 *Igfbp5* transgenic mice were scored. IENFD data are presented and statistically analyzed as the mean number of nerve fibers per mm^2^ of epidermis. For visual presentation, linear brightness and contrast correction was performed consistently in the corresponding images without changing image information.

### Electrophysiology

Analyses of nerve conduction studies were initially performed with a Toennies electromyograph (Toennies, Würzburg) and later with a digital Neurosoft-Evidence 3102 electromyograph (Schreiber & Tholen Medizintechnik). Motor and compound sensory–motor nerve conduction was investigated in the sciatic nerve in mutant and control mice using techniques described previously for numerous mutant mouse lines [6, 29, 57]. For details see supplementary methods section.

### Statistical analysis

All data are expressed as mean ± SD (*P < 0.05, **P < 0.01, ***P < 0.001). For statistical analysis, 3 independent experiments were conducted. After testing for normal distribution, the data were subjected to a statistical analysis with two-tailed Student’s *t* test when comparing two groups and one-way ANOVA with the Tukey post hoc test for comparison of more than 2 groups. The frequency distribution of axon circumferences was analyzed by two-way ANOVA with the Bonferroni post hoc test. Statistical analysis was performed with Graph Pad Prism4 Software (San Diego, USA). Final figures were arranged using ImageJ, Adobe Photoshop and Adobe Illustrator CS6.

## Results

### IGFBP5 protein levels are elevated in nerve biopsies in DNP

**Fig. 1:**
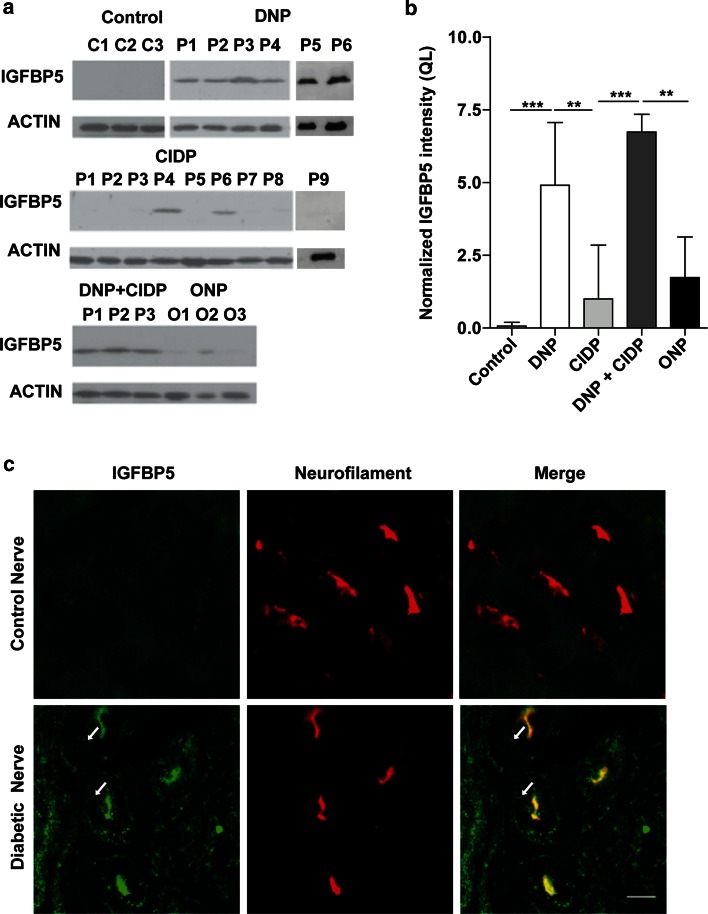
IGFBP5 protein levels are significantly up-regulated in biopsies of DNP patients. **a** Western blots of IGFBP5 protein levels in human sural nerve from control (C) individuals (3 samples shown from a total of 5 analyzed), patients (P) with DNP, CIDP, DNP+CIDP (3 samples shown from 4 in total) and other neuropathies (ONP; axonal neuropathy in motor neuron disease (O1, O3), vitamin B12 deficiency (O2). **b** IGFBP5 protein levels were specifically up-regulated in patients with DNP. Biopsies of patients with DNP showed on average a 5-fold up-regulation compared to CIDP individuals and 50-fold in comparison to healthy controls. Up-regulation in DNP patients with additional CIDP was not significantly higher than in patients with DNP alone. Other neuropathies (ONP) and patients with CIDP showed no significant change in comparison to controls. All values were normalized to CIDP biopsies. QL: quantitative labeling. **c** IGFBP5 (*green*) and neurofilament (*red*) distribution in human sural nerves from a control individual and a patient with DNP. Note that IGFBP5 levels were increased in axons and in the extracellular matrix (ECM) (*arrows*) in DNP. *Scale bar* 5 μm

Previous studies have shown that IGF1 levels are down-regulated in peripheral nerves in diabetic [53] and non-diabetic [16] types of neuropathy. We therefore investigated the expression of IGF family members, their receptors and IGFBPs by microarray expression analysis of sural nerve biopsies from patients with DNP and age-matched control individuals (electronic supplementary Table A1). Microarray expression analysis revealed a decrease of *IGF1* mRNA levels and more than sevenfold up-regulation of IGFBP5 expression in DNP. These findings were corroborated by Western blotting using protein extracts from sural nerve biopsies (Fig. 1a). IGFBP5 protein levels were more than 5-fold up-regulated in 6 patients with DNP (*P* < 0.01, one-way ANOVA) (see electronic supplementary Table 2 for clinical characteristics of these patients) and in 3 patients with combined diabetic neuropathy and chronic inflammatory demyelinating polyradiculoneuropathy (DNP+CIDP; *P* < 0.001, one-way ANOVA) as compared to CIDP individuals (Fig. 1b, c) and 50-fold in comparison to healthy controls (*P* < 0.001, one-way ANOVA). Increased IGFBP5 protein levels were only observed in 2 out of 9 CIDP patients, and did not reach statistical significance when compared with controls (*P* > 0.05, one-way ANOVA). This suggests an association of IGFBP5 overexpression with DNP rather than with the superimposed CIDP in this mixed group. IGFBP5 expression was also low in the 3 patients with non-diabetic neuropathy (*P* > 0.05, one-way ANOVA) (Fig. 1a, b). This indicates that levels of IGFBP5 are specifically up-regulated in patients with DNP. Enhanced levels of IGFBP5 could also be detected by immunohistochemistry in peripheral nerve sections (Fig. 1c), particularly in axons, as revealed by co-localization with neurofilament H immunoreactivity. IGFBP5 immunoreactivity was also observed in the extracellular matrix surrounding the axons (arrows in Fig. 1c).

### IGFBP5 inhibits motoneuron survival and axon growth

**Fig. 2:**
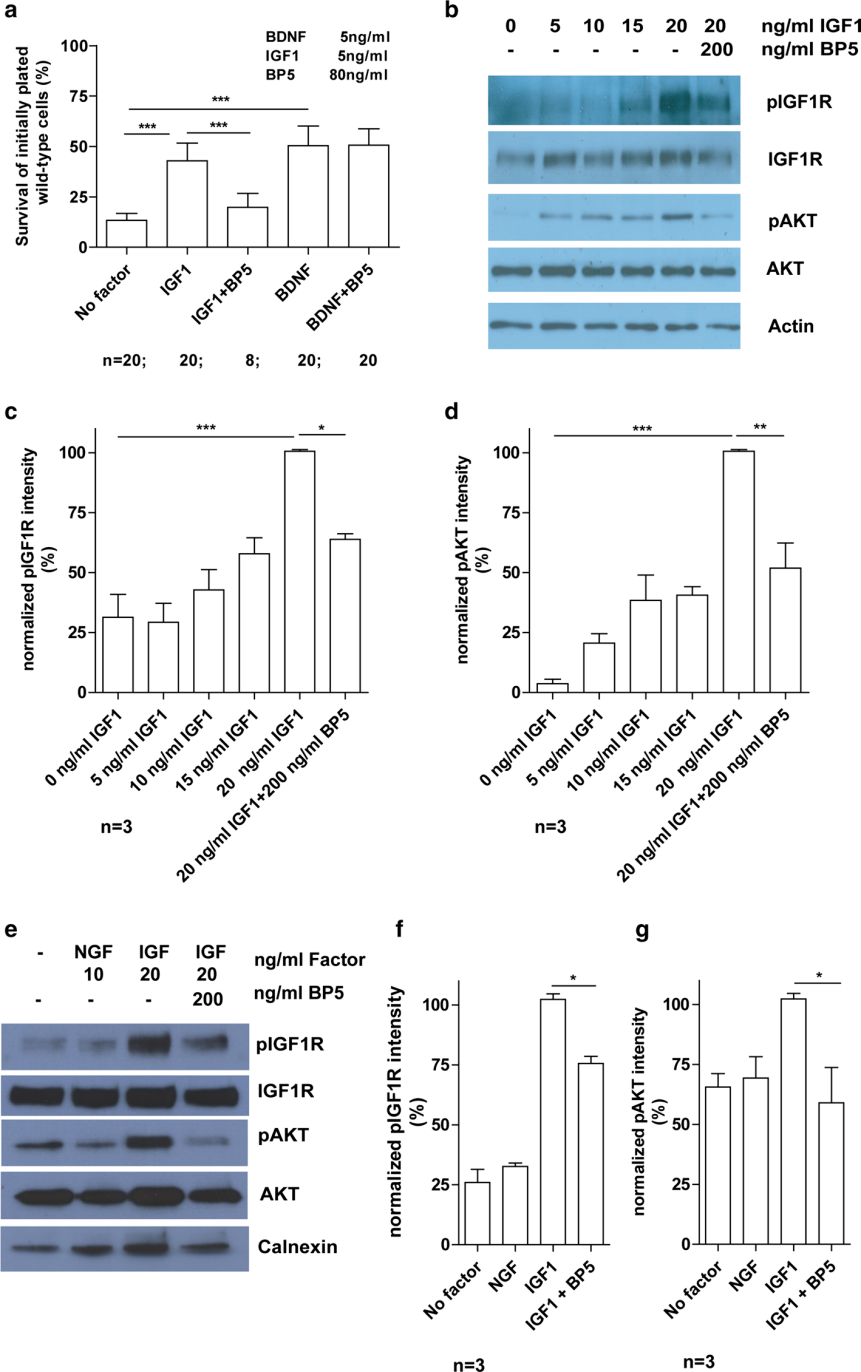
IGFBP5 decreases the survival promoting downstream effect of IGF1 on isolated wild-type motoneurons. **a** IGFBP5 (BP5) reduced IGF1-mediated survival effects from 43 to 20 % on wild-type motoneurons after 7 days in culture, while IGFBP5 did not inhibit BDNF-mediated survival. **b** Downstream activation of IGF1 signaling pathway in wild-type motoneurons after stimulation with different IGF1 concentrations. Wild-type motoneuron cell cultures were grown for 5 days with 5 ng/ml BDNF, starved overnight and pulsed for 20 min with indicated IGF1 concentrations. Western blot analyses revealed the strongest phosphorylation of IGF1R (**b**, **c**) and AKT (**b**, **d**) with 20 ng/ml IGF1. **e** Downstream activation of IGF1 signaling pathway in wild-type DRGs after stimulation with IGF1. Wild-type DRG cell cultures were grown for 20 h with 10 ng/ml NGF, starved for 4 h and pulsed for 20 min with indicated factors. Western blot analyses revealed the strongest phosphorylation of IGF1R (**e**, **f**) and AKT (**e**, **g**) with 20 ng/ml IGF1

Based on the significant up-regulation of IGFBP5 expression in nerves of diabetic patients, we investigated whether IGFBP5 interferes with the effects of IGF1 on cultured spinal motoneurons, grown for 7 days with IGF1 or BDNF (5 ng/ml) alone, or in combination with IGFBP5 (80 ng/ml). IGF1 and BDNF had potent effects on motoneuron survival (*P* < 0.001, one-way ANOVA). IGF1 maintained 43 % of initially plated motoneurons; BDNF maintained 50 % (Fig. 2a). Addition of IGFBP5 (80 ng/ml) reduced IGF1-mediated survival rates to 20 % (*P* < 0.001, one-way ANOVA), whereas BDNF-mediated survival rates remained unaffected (*P* > 0.05, one-way ANOVA) (Fig. 2a). We then tested the phosphorylation of the IGF1R and downstream AKT with increasing IGF1 concentrations (Fig. 2b). We found maximal activation of the receptor and phosphorylation of downstream AKT with 20 ng/ml IGF1 (*P* < 0.001, one-way ANOVA) (Fig. 2b–d). Addition of IGFBP5 (200 ng/ml) led to significant reduction of IGF1R (*P* < 0.05, one-way ANOVA), and downstream AKT phosphorylation (*P* < 0.01, one-way ANOVA), indicating that IGFBP5 is inhibitory for IGF1R activation and survival in cultures of isolated motoneurons (Fig. 2b–d). Similar observations were made with sensory neurons from embryonic lumbar DRGs (Fig. 2e–g). IGFBP5 reduced IGF-mediated activation of the IGF1R (*P* < 0.05, one-way ANOVA) and AKT (*P* < 0.05, one-way ANOVA). AKT activation by NGF was not affected.

**Fig. 3:**
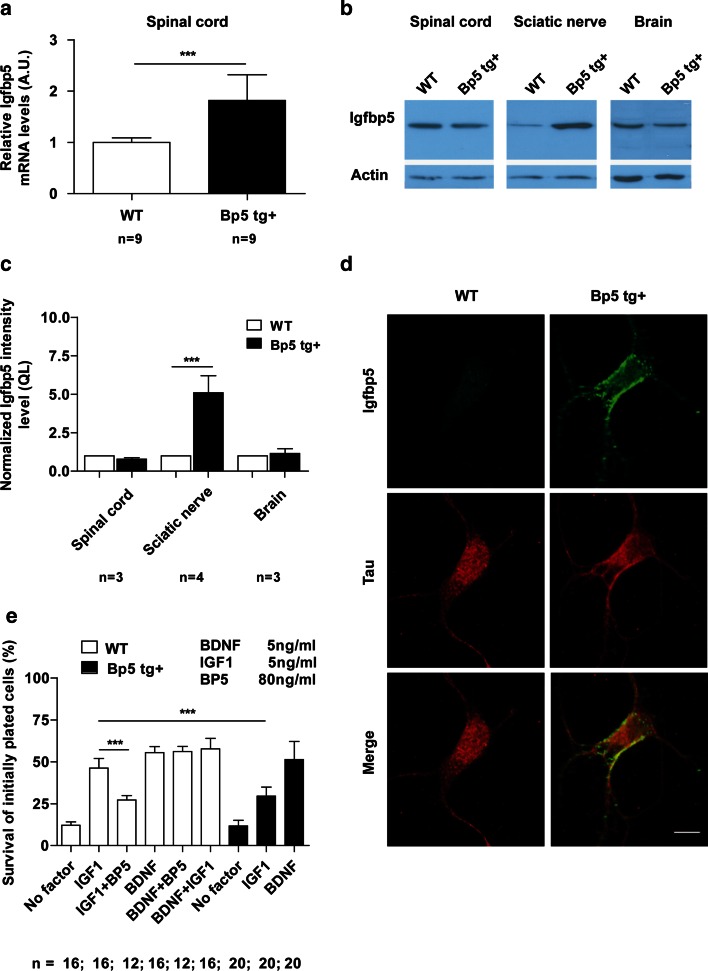
*Igfbp5 tg*+ motoneurons show decreased survival when cultured with IGF1. **a** qRT-PCR revealed a 1.8-fold increase of the *Igfbp5* mRNA level in the spinal cord of 6-month-old *Igfbp5 tg*+ mice (*Bp5 tg*+). Scored values were obtained by normalizing to β-actin mRNA levels. *A.U.* arbitrary units. **b** Igfbp5 protein elevation was detected in sciatic nerve, but not in spinal cord and brain of 6-month-old *Igfbp5 tg*+ mice compared to controls. Actin was used as loading control. **c** Quantification of Igfbp5 protein levels. *QL* quantitative labeling. **d** Motoneurons stained for Tau (*red*) and Igfbp5 (*green*) 7 days after plating. Igfbp5 was increased in the soma and neurites of *Igfbp5 tg*+ motoneurons. Igfbp5 appeared associated with the cell membrane. *Scale bar* 5 μm. **e** The positive effect of IGF1 on motoneuron survival is reduced by 37 % in *Igfbp5 tg*+ motoneurons. Motoneuron survival was unchanged in the presence of BDNF

**Fig. 4:**
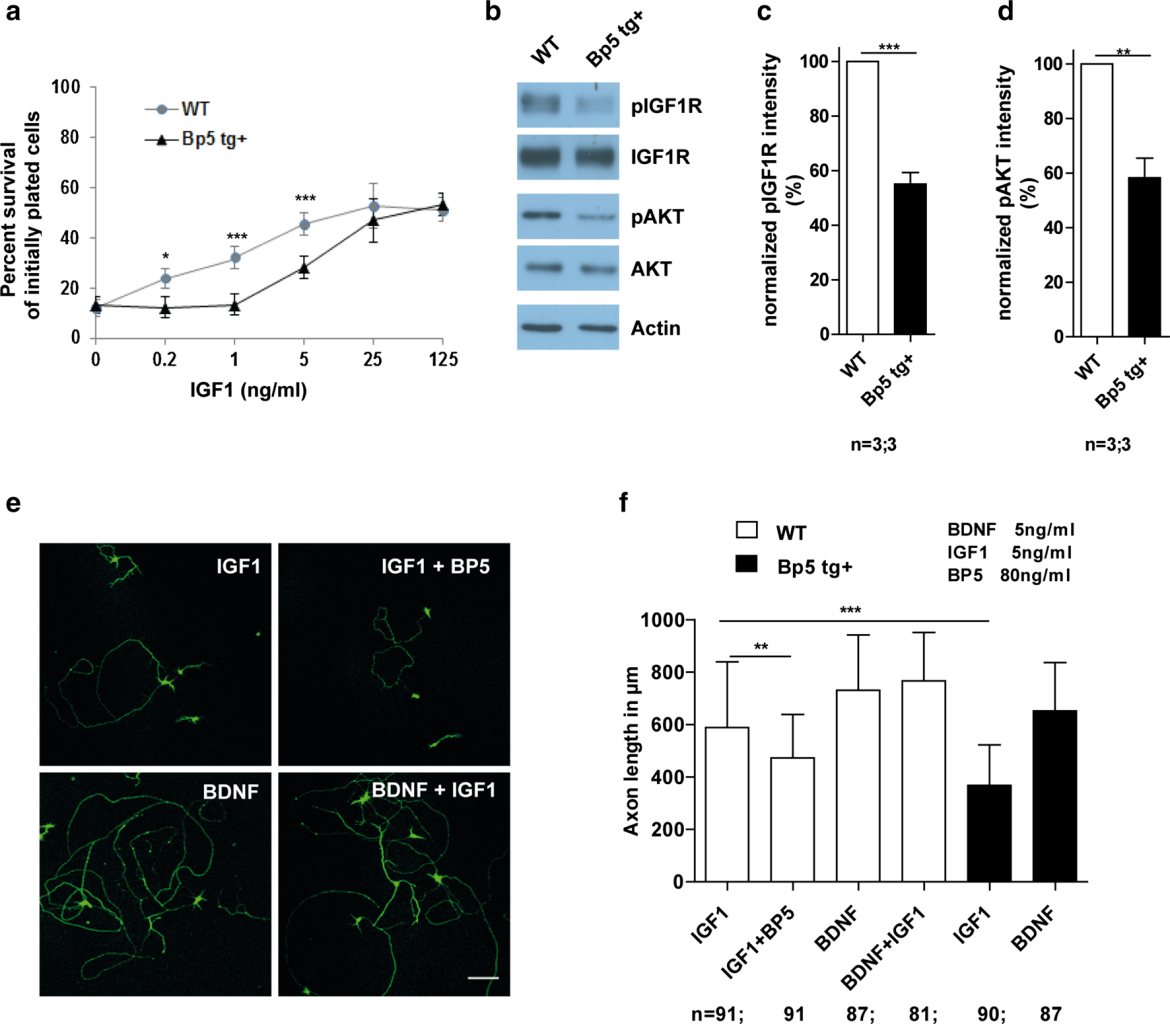
Reduced phosphorylation of IGF1 receptor in *Igfp5 tg*+ motoneurons leads to reduced survival and axon outgrowth. **a** Dose–response curve for IGF1-mediated motoneuron survival. Wild-type and *Igfp5 tg*+ motoneurons (7 DIV) were treated with different IGF1 concentrations. **b** Reduced phosphorylation of IGF1 receptor and AKT in *Igfbp5 tg*+ motoneurons compared to wild-type motoneurons. **c** Quantification of pIGF1R protein levels in motoneurons. **d** Quantification of pAKT protein levels in motoneurons. **e** Images of cultured wild-type motoneurons stained against Tau to identify axons. *Scale bar* 100 μm. **f** Igfbp5 (BP5) inhibited IGF1-induced axon outgrowth in wild-type motoneurons. *Igfbp5 tg*+ motoneurons showed shorter axons than wild-type axons when cultured with IGF1. Axon growth stimulated by BDNF was not affected in *Igfbp5 tg*+ motoneurons

To study this effect on motoneurons and sensory neurons in vivo, we generated mice with neuron-specific overexpression of IGFBP5. An 8-kb fragment including the human *NF*-*L* promoter, mouse *Igfbp5* cDNA, the polyA signal from pMC-Cre and exons 2–4 of the *NF*-*L* gene was injected into fertilized mouse eggs (for details see electronic supplementary Fig. A1, supplementary methods section). Real-time RT-PCR analysis revealed that the levels of *Igfbp5* mRNA in the spinal cord of 6 months old *Igfbp5* transgenic mice (*Bp5 tg*+) were up-regulated 1.8-fold compared with controls (*P* < 0.001, two-tailed Student’s *t* test) (Fig. 3a). Western blot analysis for IGFBP5 in the spinal cord, sciatic nerve and brain of 6-month-old *Igfbp5* transgenic mice exhibited a fivefold IGFBP5 protein up-regulation in the sciatic nerve (*P* < 0.001, two-tailed Student’s *t* test) (Fig. 3b, c), indicating that the IGFBP5 protein is anterogradely transported in axons. Isolated motoneurons from *Igfbp5* transgenic embryos (E13.5) also showed increased IGFBP5 immunoreactivity in cell bodies and axons after 7 days in vitro (Fig. 3d). IGFBP5 was predominantly localized on the surface of these neurons. This correlates with previous data showing that IGFBP5 binds to the cell surface and the surrounding extracellular matrix [2]. *Igfbp5* transgenic motoneurons showed a different survival response to IGF1 than wild-type motoneurons. While survival of *Igfbp5-*overexpressing motoneurons was unchanged in the presence of BDNF (*P* > 0.05, one-way ANOVA), their survival was significantly reduced by 37 % in the presence of IGF1 compared to wild-type motoneurons (*P* < 0.001, one-way ANOVA) (Fig. 3e). When we compared the dose response for IGF1 on motoneuron survival in *Igfbp5* transgenic and wild-type motoneurons, a shift was observed (0.2 ng/ml IGF1; *P* < 0.05; 1 ng/ml IGF1; *P* < 0.001; 5 ng/ml IGF1; *P* < 0.001; one-way ANOVA) (Fig. 4a), indicating that motoneurons overexpressing IGFBP5 need more IGF1 for their survival than non-transgenic motoneurons. This result provides further evidence that IGFBP5 inhibits IGF1 actions on motoneurons. We then compared the phosphorylation levels of IGF1R and downstream AKT in *Igfbp5* transgenic motoneurons relative to wild-type controls. A strong reduction in IGF1R and AKT phosphorylation levels was observed in *Igfbp5* transgenic motoneurons compared to controls (pIGF1R: *P* < 0.001; pAKT: *P* < 0.01, two-tailed Student’s *t* test) grown for 5 days with 5 ng/ml BDNF, then starved overnight and pulsed for 20 min with 20 ng/ml IGF1 (Fig. 4b–d). Motoneurons overexpressing IGFBP5 also exhibited reduced axon growth (Fig. 4e, f), when cultured with IGF1 at a concentration of 5 ng/ml (*P* < 0.001, one-way ANOVA). Axon growth was normal when these motoneurons were cultured with 5 ng/ml BDNF (*P* > 0.05, one-way ANOVA), indicating that IGFBP5 not only reduces survival but also specifically inhibits the effects of IGF1 on axon growth.

### Neuronal IGFBP5 overexpression leads to impaired motor function, degeneration and loss of motor axons and cell bodies

**Fig. 5:**
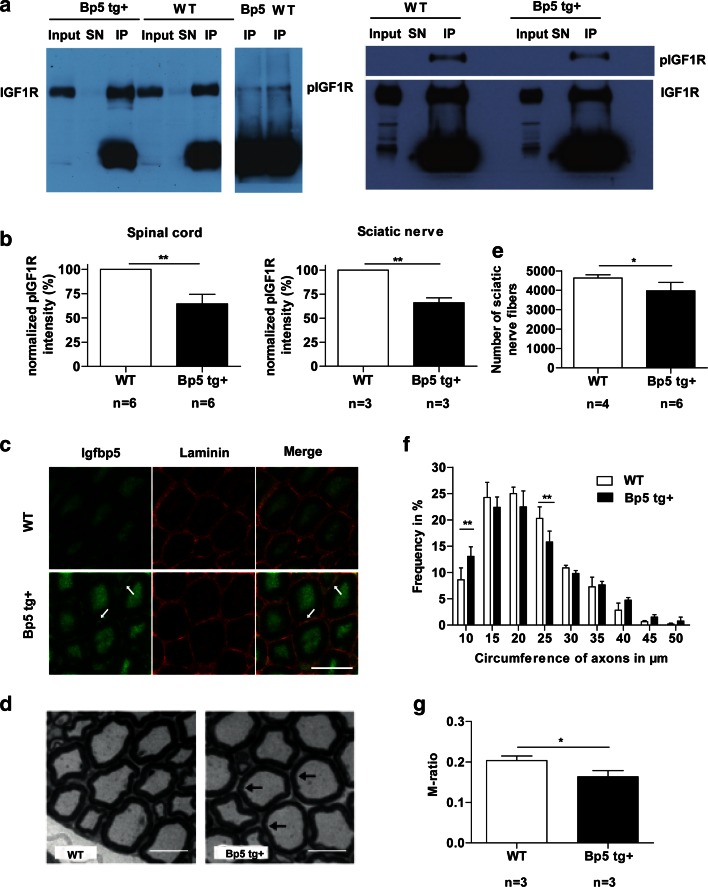
IGFBP5-overexpressing mice show decreased activation of IGF1 receptor that corresponds to motoneuron degeneration and myelination defects. **a**, **b** Immunoprecipitation of the IGF1 receptor from spinal cord (*left panel*) and sciatic nerve (*right panel*) extracts of 4-day-old mice and subsequent analysis of phosphorylation showed decreased activation levels in *Igfbp5 tg*+ mice compared to controls. *SN* supernatant, *IP* immunoprecipitation. **c** Localization of Igfbp5 in cross sections of sciatic nerves. Igfbp5 was increased in axons and extracellular matrix of *Igfbp5 tg*+ mice. *Arrows* depict extracellular matrix staining. *Scale bar* 5 µm. **d** Representative photomicrograph of myelinated fibers in semithin cross sections of the sciatic nerve of 6-month-old wild-type and *Igfbp5 tg*+ animals. *Scale bar* 10 µm. **e** 5- to 6-month-old *Igfbp5 tg*+ mice showed a 14 % loss of sciatic nerve fibers. **f** The frequency of fibers with a circumference between 20–25 μm was decreased by 4.5 % in the sciatic nerve of 6-month-old *Igfbp5 tg*+ mice. **g** The M-ratio was significantly reduced by 20 % in 6-month-old *Igfbp5 tg*+ mice

To test whether neuronal IGFBP5 overexpression reduces responsiveness to endogenous IGF1, we immunoprecipitated the IGF1R from the spinal cord and sciatic nerve of 4-day-old *Igfbp5* transgenic mice, a time period when endogenous IGF1 levels are high. Analyses of spinal cord and sciatic nerve extracts by Western blot with a specific pIGF1R antibody revealed a significant 35 % reduction in the phosphorylation of the IGF1R (*P* < 0.01, two-tailed Student’s *t* test) (Fig. 5a, b). Elevated IGFBP5 immunoreactivity was observed in axons of the sciatic nerve and the extracellular matrix of *Igfbp5* transgenic mice (see arrows in Fig. 5c), closely resembling the distribution of IGFBP5 in sural nerve biopsies of patients with DNP (Fig. 1d).

**Fig. 6:**
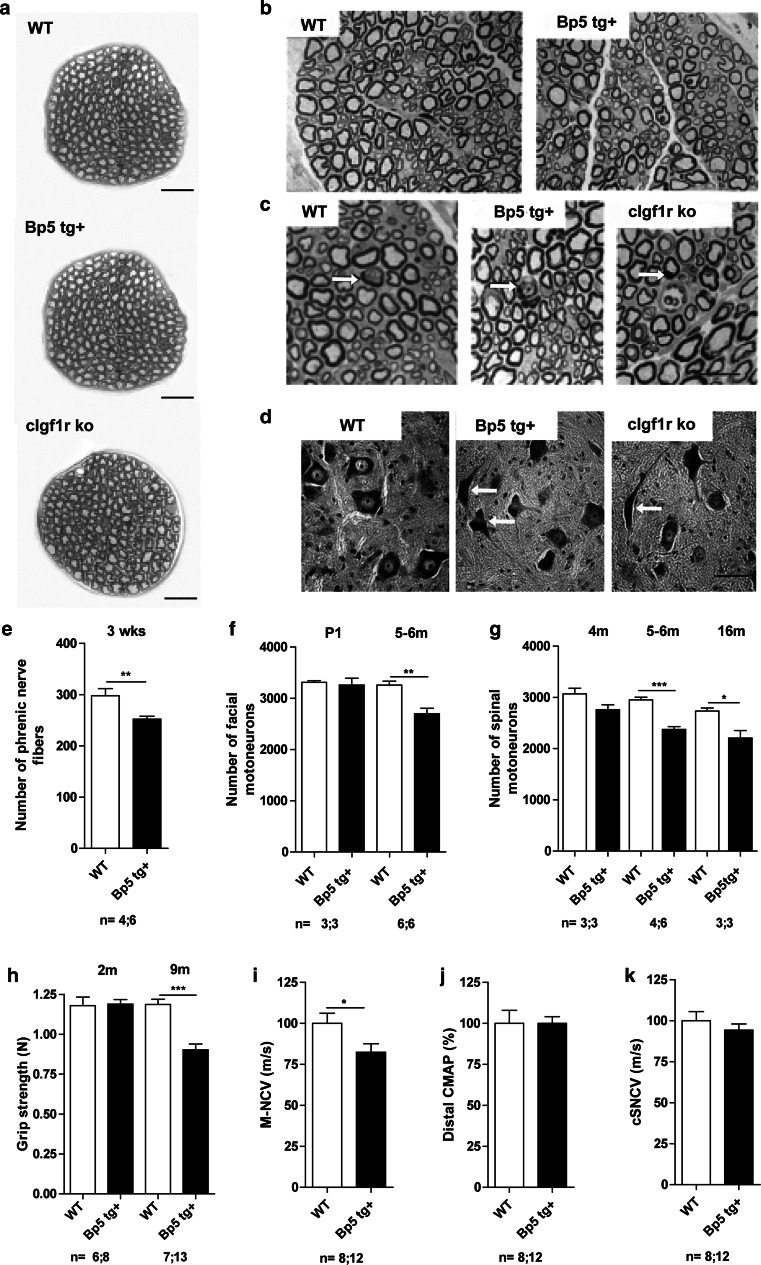
Nerve fiber degeneration and spinal motoneuron loss are partly reflected in electrophysiological alterations in motor nerves of *Igfbp5* transgenic mice. **a** Light micrographs of 6-month-old wild-type, *Igfbp5 tg*+ and *cIgf1r ko* phrenic nerve semithin sections stained with azur–methylene blue. *Scale bar* 20 µm. **b**, **c** Sciatic nerve cross sections of wild-type and *Igfbp5 tg*+ mice. No degenerating fibers were detectable in control tissue and small fibers appeared normal in *Igfbp5 tg*+ mice. Larger fibers of 6-month-old wild-type and *Igfbp5 tg*+ mice (*arrows*) showed signs of degeneration. *Scale bar* 10 μm. **d** Nissl-stained paraffin sections of the lumbar spinal cord showed degenerating motoneurons in *Igfbp5 tg*+ mice compared to wild-type animals. *Scale bar* 50 μm. **e** 3-week-old *Igfbp5 tg*+ mice showed a 15 % loss of phrenic nerve axons. **f**, **g** In 5- to 6-month-old *Igfbp5 tg*+ mice, the number of facial motoneurons was reduced by 17 %, and the number of lumbar spinal motoneurons by 20 %. **h** 9-Month-old *Igfbp5 tg*+ mice showed reduced grip strength compared with wild-type animals. **i** Motor nerve conduction velocity (M-NCV) was reduced significantly in *Igfbp5 tg*+ mice by 18 % in sciatic nerve. **j** Distal compound muscle action potential (CMAP) amplitudes were not altered in *Igfbp5 tg*+ and control animals. **k** Compound sensory–motor nerve conduction velocity (cSNCV) was not altered in *Igfbp5 tg*+ mice

Next, we investigated the morphology of peripheral nerves. The number of sciatic nerve axons in *Igfbp5* transgenic mice was reduced by 14 % when compared to wild-type animals at 6 months of age (*P* < 0.05, two-tailed Student’s *t* test) (Figs. 5e, 6b, c [arrows indicate degenerating axons], electronic supplementary Table A3). In parallel, a change in fiber size distribution in the sciatic nerve became apparent, with a significant reduction of fibers with a circumference between 20–25 µm in IGFBP5-overexpressing mice (*P* < 0.01, two-way ANOVA) (Fig. 5f). In addition, the M-ratio was significantly reduced by 20 % in transgenic animals (*P* < 0.05, two-tailed Student’s *t* test) (Fig. 5d, g). Quantification of myelinated axons in the phrenic nerve of 3-week-old *Igfbp5* transgenic mice revealed a significant axonal loss of 15 % (*P* < 0.01, two-tailed Student’s *t* test) (Fig. 6e; electronic supplementary Table A3). No further loss of phrenic nerve fibers was detected in 5- to 6-month-old *Igfbp5* transgenic mice (electronic supplementary Table A3; Fig. 6a). These data suggest that local inhibition of IGF1 not only affects motor axons but also the degree of myelination in Schwann cells. To determine whether axon degeneration and axon loss reflected loss of motoneuron cell bodies, we also counted motoneurons in the facial nucleus and the lumbar spinal cord of wild-type and *Igfbp5* transgenic mice at different stages of postnatal development. In newborn and 3-week-old animals (Fig. 6f), numbers of facial motoneurons were not significantly different between wild-type and *Igfbp5* transgenic mice (*P* > 0.05, one-way ANOVA) (electronic supplementary Table A3), indicating that neuronal IGFBP5 overexpression has no effect on the generation and developmental cell death of motoneurons. However, by 5–6 months of age, a 17 % loss of facial motoneurons became apparent (*P* < 0.01, one-way ANOVA) (Fig. 6f; electronic supplementary Table A3). In the spinal cord, no significant loss of motoneurons could be observed at 4 months of age (*P* > 0.05, one-way ANOVA). In contrast, at 5–6 months, a 20 % loss of motoneurons was observed (*P* < 0.001, one-way ANOVA) (Fig. 6g, electronic supplementary Table A3) and remaining spinal motoneurons exhibited signs of atrophy (Fig. 6d, indicated by white arrows). At 16 months, the level of motoneuron loss remained at 20 %, indicating that spinal motoneuron loss was maximal by 5–6 months and did not progress thereafter (*P* < 0.05, one-way ANOVA) (Fig. 6g; electronic supplementary Table A3). Nine-month-old *Igfbp5* transgenic mice showed a significant reduction of forelimb grip strength (*P* < 0.001, one-way ANOVA) (Fig. 6h). In 5- to 6-month-old *Igfbp5* transgenic mice, nerve conduction studies of the sciatic nerve [6, 29, 57] revealed that motor nerve conduction velocities (M-NCVs) were reduced by 18 % when compared to controls (*P* < 0.05; two-tailed Student’s *t* test) (Fig. 6i), which is compatible with a mild, putatively secondary demyelinating component of the neuropathy. Amplitudes of distal compound muscle action potentials (CMAPs) and compound sensory–motor nerve conduction velocities (cSNCVs) were not significantly altered (*P* > 0.05; two-tailed Student’s *t* test) (Fig. 6j, k; electronic supplementary Fig. A1).

### Sensory defects in *Igfbp5* transgenic mice

Since sensory defects are a hallmark of diabetic neuropathy, we studied a sensory nerve (saphenous nerve) in IGFBP5-overexpressing mice in more detail and also tested behavioral parameters of sensory function. The gross overall morphology of the saphenous nerve appeared unaltered in 10–11 month-old *Igfbp5* transgenic mice compared with that of age-matched wild-type mice (electronic supplementary Fig. A2). Nevertheless, 10–11 month-old *Igfbp5* transgenic mice demonstrated a higher withdrawal threshold in the von Frey hair stimulation test (*P* < 0.01, two-tailed Student’s *t* test) and thermal hypoalgesia test as illustrated by longer withdrawal latencies to a noxious heat stimulus in the Hargreaves test (*P* < 0.001, two-tailed Student’s *t* test) (electronic supplementary Fig. A3). This indicates that sensory neurons are also affected in *Igfbp5* transgenic mice. In addition, deficits in coordination and balance were revealed on an accelerating rotarod. 9-Month-old *Igfbp5* transgenic mice fell from the device in significantly less time than control animals (*P* < 0.001, one-way ANOVA) (electronic supplementary Fig. A3), whereas younger 2-month-old mice did not show any deficits in this test.

In the glabrous footpad skin, almost all nerve fibers in the epidermis are unmyelinated free nerve endings [58] and can be visualized using the pan-neuronal marker PGP9.5. The density of PGP9.5-positive fibers which passed through the basement membrane of the epidermis appeared significantly reduced in 10-month-old *Igfbp5* transgenic mice (*P* < 0.01, two-tailed Student’s *t* test) (electronic supplementary Fig. A3). In line with this observation, reduced substance P levels were found in the footpad of 10-month-old *Igfbp5* transgenic mice compared with control animals (*P* < 0.05, two-tailed Student’s *t* test) (electronic supplementary Fig. A3).

### Conditional deletion of IGF1 receptor in neurons results in axonopathy and motoneuron loss similar to that seen with IGFBP5 overexpression

To investigate whether degeneration of motoneurons in *Igfbp5* transgenic mice is caused primarily by the reduced availability of IGF1 for axons, or whether this is secondary to effects on Schwann cells, we analyzed mice lacking the type 1 IGF receptor in neurons (*cIgf1r ko*). For this purpose, mice carrying loxP sites flanking exon III of the mouse *Igf1r* gene were generated (for details see electronic supplementary Fig. A4; supplementary methods section). Exon III encodes most of the cysteine-rich ligand-binding domain of the α-subunit of the receptor, and disruption of this exon generates an inactive receptor [30]. The Cre recombinase is controlled by the NF-L promoter which has been shown to be highly selective for neurons [41]. Neuron-specific disruption of exon III was confirmed by RT-PCR (electronic supplementary Fig. A4). Immunoprecipitation of the IGF1R from spinal cord of 4-day-old *cIgf1r ko* mice revealed a 32 % reduction in IGF1R phosphorylation in the spinal cord (electronic supplementary Fig. A4). Histological analysis of the sciatic nerve of *cIgf1r ko* mice revealed a 13 % reduction of nerve fibers at 6 months (*P* < 0.05; two-tailed Student’s *t* test) (Fig. 6c; electronic supplementary Fig. A4, electronic supplementary Table A4). As found in IGFBP5-overexpressing mice, *cIgf1r ko* mice showed a decrease of 6 % in fibers with a circumference between 20–25 μm (*P* < 0.05, two-way ANOVA) (electronic supplementary Fig. A4). As expected, the M-ratio was normal (*P* > 0.05, two-tailed Student’s *t* test) (electronic supplementary Fig. A4), because IGF1 signaling in Schwann cells was unaffected by neuron-specific inactivation of the mouse *Igf1r* gene. The number of myelinated axons was also reduced by 11 % in the phrenic nerve of *cIgf1r ko* mice (*P* < 0.05, two-tailed Student’s *t* test) (Fig. 6a; electronic Table A4). In 9-month-old *cIgf1r ko* mice, a significant reduction of motoneurons by 16 % was observed in the facial nuclei (*P* < 0.05, two-tailed Student’s *t* test) and by 21 % in the lumbar spinal cord (*P* < 0.01, two-tailed Student’s *t* test) compared to controls, an effect that was not observed in newborn mice (electronic supplementary Fig. A5 and Table A4). In addition, atrophic and degenerating motoneurons were frequently found in the spinal cord (Fig. 6d, indicated by white arrows). We then investigated potential electrophysiological alterations. Comparison of wild-type and *cIgf1r ko* mice revealed that distal compound motor action potentials (CMAPs), motor nerve conduction velocities (M-NCVs) and compound sensory–motor nerve conduction velocities (cSNCVs) were not significantly reduced in *cIgf1r ko* mice (*P* > 0.05, two-tailed Student’s *t* test) (electronic supplementary Fig. A5). A mild abnormality, namely increased dispersion in the F-waves (late responses traveling to and returning from the anterior horn cells along the entire length of the peripheral nerve fibers), was observed (*P* < 0.05, two-tailed Student’s *t* test) (electronic supplementary Fig. A5). This finding may be an indicator that some F-wave generating individual nerve fibers display minor conduction abnormalities at some level along their axons. These changes are compatible with, but not specific for, a mild axonal pathology [21, 57] and resemble features found in some patients with very early DNP [36, 48].

## Discussion

Here, we show that IGFBP5 is more than 7-fold up-regulated at the mRNA level and at least 5-fold at the protein level in diabetic nerves compared to nerves from CIDP individuals and 50-fold compared to nerves from non-diabetic control individuals suggesting a pathogenic role of IGFBP5 in diabetic neuropathy. IGFBP5 is mainly found in axons and within the extracellular matrix surrounding the axons, indicating that it is produced in motoneurons, anterogradely transported and released into the extracellular space. Thus, it could interfere with the signaling of IGF1 derived both from the circulation and from contacting Schwann cells. Both Schwann cells and axons express IGF1 receptors, and altered IGF1 responses therefore could affect both axon and Schwann cell integrity. To test this hypothesis, two new mouse models were generated, with overexpression of IGFBP5 in motoneurons, and specific ablation of the IGF1R in the same population of cells. Our results show that elevated IGFBP5 protein levels and reduced IGF1 signaling in peripheral nerves are important factors contributing to progressive loss of motor fibers and subsequent loss of motoneurons, and they are also associated with classical signs of diabetic neuropathy such as altered thresholds for heat, pain and mechanosensation. Previous studies have reported reduced levels of circulating IGF1 as a common feature in diabetes, with the highest reductions seen at older ages and with longer duration of disease [47]. Similarly, in the streptozotocin-induced rat model, reduced IGF1 expression in peripheral nerves is observed at early stages of disease [53]. Our data indicate that additional components of IGF1 signaling are dysregulated in the peripheral nervous system. The IGF1R that mediates the trophic effects of IGF1 on Schwann cells and neurons is up-regulated in nerve biopsies from patients, as is IGFBP5, an IGF1-binding protein that inhibits IGF1-mediated survival and axon growth in motoneurons. IGF1 depletion in mice leads to a broad range of defects in the brain, with hypomyelination and loss of some neuronal populations in the CNS [3]. These mice die by about 2 months of age. Postnatally, they do not exhibit any significant loss of motoneuron cell bodies at early stages, indicating that motoneuron development during embryogenesis does not depend on IGF1. Similarly, mice overexpressing IGFBP5 appear normal at birth and at younger ages; however, by 6 months of age, prominent dysmyelination occurs. Similarly, the loss of motor fibers and motoneuron cell bodies was not detectable until 6 months of age, a stage that IGF1-deficient mice do not reach [3]. Interestingly, dysmyelination was not observed in *cIgf1r ko* mice, indicating that the observed defects in peripheral nerve myelination in IGFBP5-overexpressing mice are likely to be due to reduced IGF1 function in Schwann cells. These conditional IGF1R deletion mutants further revealed that axon maintenance is directly dependent on IGF1R activation, since loss of large motor fibers in the phrenic, facial, and the mixed sciatic nerve was observed in the *cIgf1r ko* mice. Sensory axons appeared affected too, reflected by altered responses to heat and mechanical stimulation in *Igfbp*5 transgenic animals. Furthermore, *Igfbp*5 transgenic mice show reduced latency to fall from an accelerating rotarod, pointing to the involvement of proprioceptive neurons. Together with the altered thresholds for heat-sensing thermoreceptors, reduced substance P levels and reduced density of small fibers in the skin, this resembles classical signs of diabetic neuropathy, which affects both sensory neurons and motor neurons, the last named predominantly at later stages. Thus, the reduced availability of IGF1 coupled with elevated expression of IGFBP5 in diabetic nerves would be predicted to have a major effect on myelination and trophic support of axons.

Up-regulation of IGFBP5 has also been observed in other organs in diabetes. The mechanisms are not clear. It has been suggested that the IGFBP5 up-regulation is induced by elevated glucose [43] and reduced insulin signaling. It could reflect a condition in diabetes under which cells protect themselves against signals for growth and mitosis when they are metabolically dysregulated. IGF1 is a potent growth factor which mediates the cellular effects of growth hormone, but it also regulates maintenance and dynamics of axon growth [7, 8], and therefore the neuronal up-regulation of IGFBP5 which appears as a protective mechanism to prevent cell growth under the metabolic conditions of diabetes also prevents the IGF-1 signaling from Schwann cells which is necessary for axon stability, axon dynamics and excitability of sensory neurons [51]. In diabetic rats, IGFBP5 mRNA up-regulation has been observed in the kidney [9, 35], suggesting that elevated IGFBP5 expression levels could contribute to kidney dysfunction during the course of diabetes. Cell cultures of cardiac fibroblasts react to elevated glucose with up-regulation of IGFBP5 protein levels, which lead to increased c-Jun N-terminal kinase phosphorylation and induction of apoptosis in cardiomyocytes [43]. This mechanism could contribute to deterioration of heart function in diabetes. Furthermore, elevated protein levels of IGFBP5 and reduced protein levels of IGF1 are found in young patients with type I diabetes showing decreased bone mass [32]. Increased levels of IGFBP5 correlate with defects in microvascular endothelial cells in retina and kidney [17], and it has been proposed that these defects could be mediated through altered response of these cells to IGF1. Our data showing that IGF1R activation is reduced in peripheral nerves from mice overexpressing IGFBP5, and that mice lacking IGF1R signaling in motoneurons show similar signs of motor neuropathy as IGFBP5-overexpressing mice strongly suggest that the up-regulation of IGFBP5 is also responsible for microangiopathy, heart failure, kidney failure, reduced bone mass and other pathological findings associated with diabetes.

Our data from mouse models suggest that inhibiting the up-regulation of IGFBP5 expression in peripheral nerves might prevent or slow down disease progression. Similarly, inhibitors of IGFBP5 that block binding to IGF1 could be of therapeutic benefit. As shown by X-ray crystallography [56], the domains of IGF1 that bind to IGFBP5 are different from those that bind to the receptor. Thus, it should be feasible to discover inhibitors that prevent the interaction of IGF1 with IGFBP5, but not with the IGF1 receptor. Such molecules could not only prevent the loss of motor axons and motor function, but also the loss of myelinated sensory axons and thus would open new strategies for testing in prospective clinical trials for DNP.

## Electronic supplementary material

Supplementary material 1 (PDF 3138 kb)
