# Supplementary material for: Dysregulated IGFBP5 expression causes axon degeneration and motoneuron loss in diabetic neuropathy
Source: Acta Neuropathol. 2015 May 30;130(3):373–87. doi: 10.1007/s00401-015-1446-8 (PMC4541707; doi:10.1007/s00401-015-1446-8)
Supplement: Supplementary file 1 — Supplementary material 1 (PDF 3138 kb) [file 401_2015_1446_MOESM1_ESM.pdf]

Supplementary Material

Supplementary Figures

Fig. A1

a

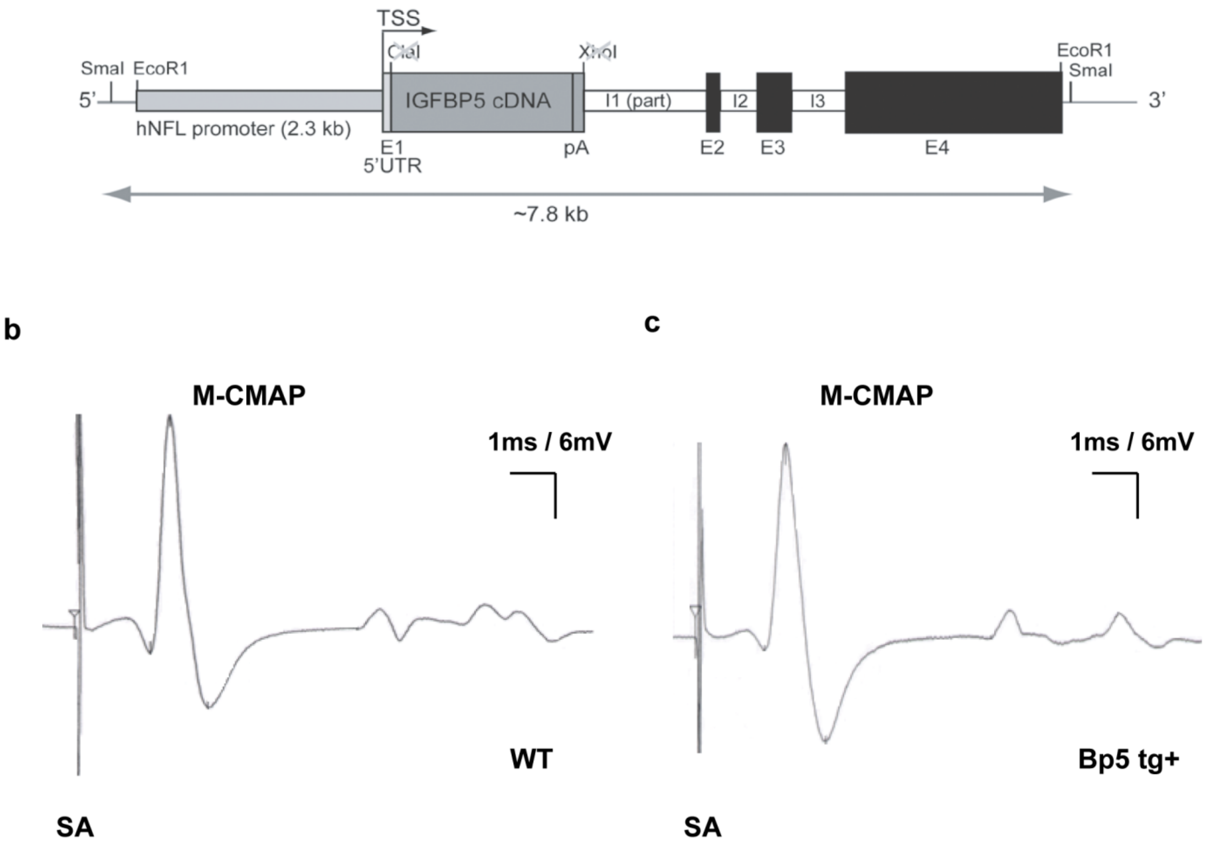

Fig. A1

a Construct for IGFBP5 overexpression under control of a neurofilament light chain promoter. **b c** Representative examples of compound motor action potentials (M-CMAP) in the sciatic nerve of 5- to 6-month-old wild-type (**b**) and *Igfbp5 tg+* mice (**c**). SA: Stimulus artefact.

Fig. A2

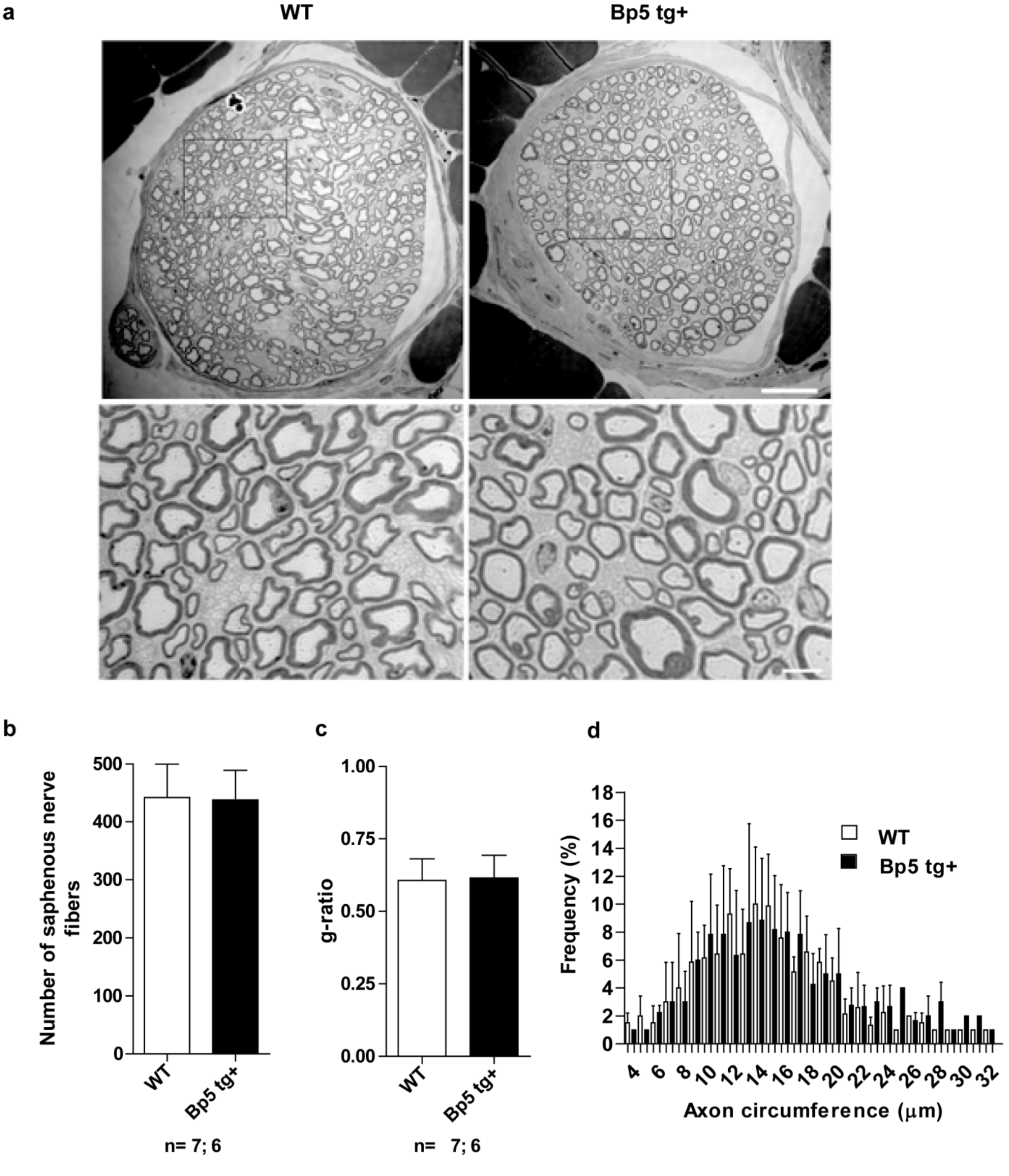

Fig. A2 Comparative analysis of the saphenous from wild-type and *Igfbp5 tg+* mice

a High resolution transmission scan overview image of wild-type and *Igfbp5 tg +* saphenous nerve sections from 10- to 11-month-old mice. Scale bar 25 µm. **b** High resolution transmission scan image of wild-type and *Igfbp5 tg+* mice. Scale bar 5 µm **c d** Axonal numbers and g-ratio in the saphenous nerve of *Igfbp5 tg+* mice were not significantly

different from wild-type. **e** Frequency histogram indicates a unimodal pattern in both wild-type and *Igfbp5 tg+* animals. All results are presented as mean ± SD determined from the analysis of *n* mice per genotype, as indicated (*$P < 0.05$, **$P < 0.01$, and ***$P < 0.001$). The white bars represent wild-type and the black bars represent *Igfbp5 tg+* mice.

Fig. A3

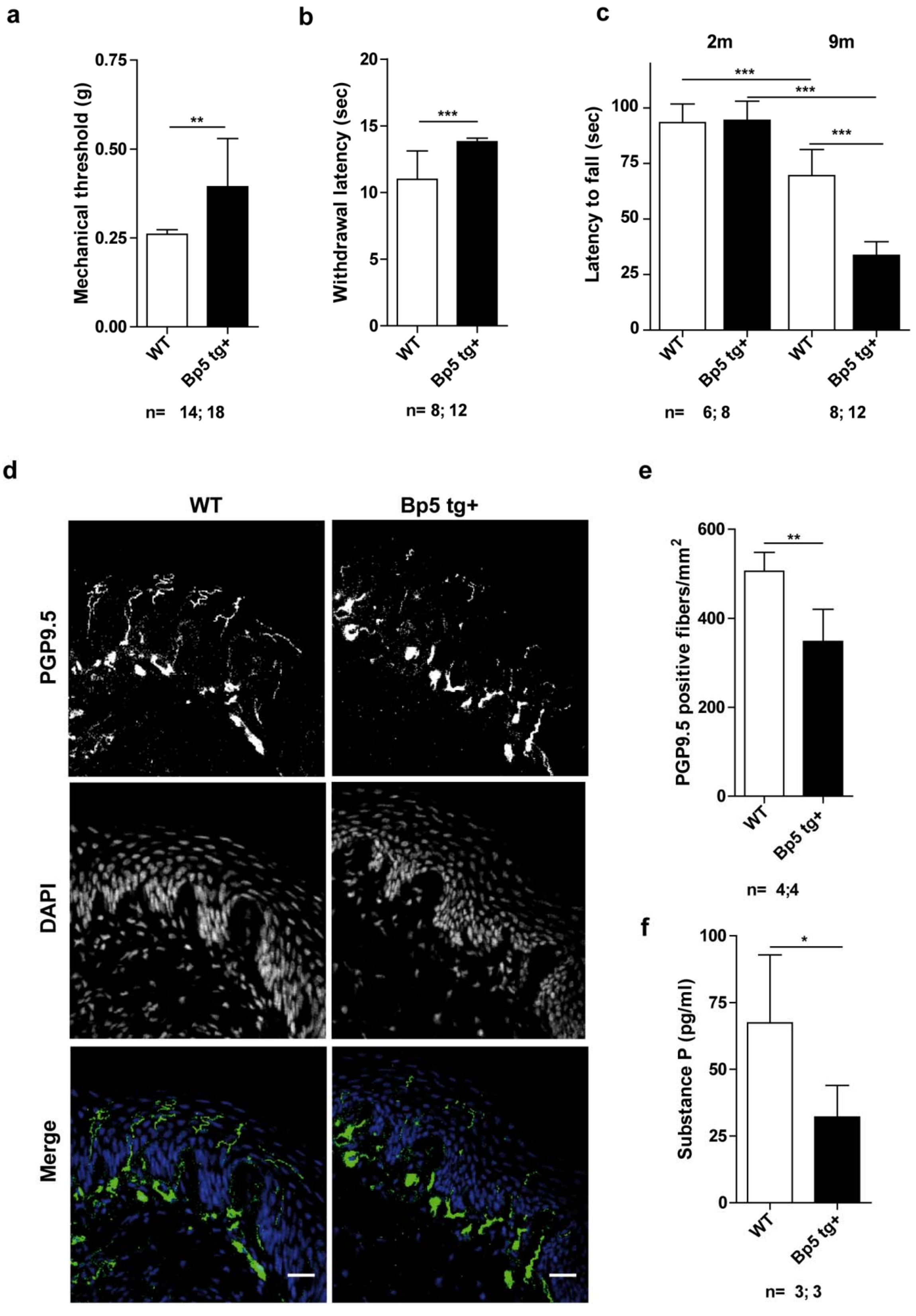
a
Mechanical threshold (g)
0.75
0.50
0.25
0.00
**
WT
Bp5 tg+
n= 14; 18
b
Withdrawal latency (sec)
20
15
10
5
0
***
WT
Bp5 tg+
n= 8; 12
c
2m
9m
Latency to fall (sec)
100
75
50
25
0
***
***
***
WT
Bp5 tg+
WT
Bp5 tg+
n= 6; 8
8; 12
d
WT
Bp5 tg+
PGP9.5
DAPI
Merge
e
PGP9.5 positive fibers/mm2
600
400
200
0
**
WT
Bp5 tg+
n= 4;4
f
Substance P (pg/ml)
100
75
50
25
0
*
WT
Bp5 tg+
n= 3; 3

Fig. A3 Small fiber function is altered in *Igfbp5 tg+* mice

a *Igfbp5 tg+* mice displayed increased mechanical paw withdrawal thresholds in the von Frey test compared with wild-type mice. **b** *Igfbp5 tg+* mice displayed significantly longer withdrawal latencies to a noxious heat stimulus in the Hargreaves test compared with wild-type. **c** Motor performance was significantly decreased in *Igfbp5 tg+* mice compared with wild-type controls on an accelerating rotarod. **d e** Intraepidermal nerve fiber density was significantly reduced in *Igfbp5* transgenic mice compared to control animals. PGP9.5 positive nerves fibers on wild-type and *Igfbp5 tg+* footpad sections are shown **(d)**. **f** Footpad substance P levels of *Igfbp5* transgenic mice were significantly reduced compared to wild-type controls. All results are presented as mean ± SD determined from the analysis of *n* mice per genotype (*$P < 0.05$, **$P < 0.01$, and ***$P < 0.001$). The white bars represent wild-type and the black bars represent *Bp5 tg+* mice. Scale bar 20 µm.

Fig. A4

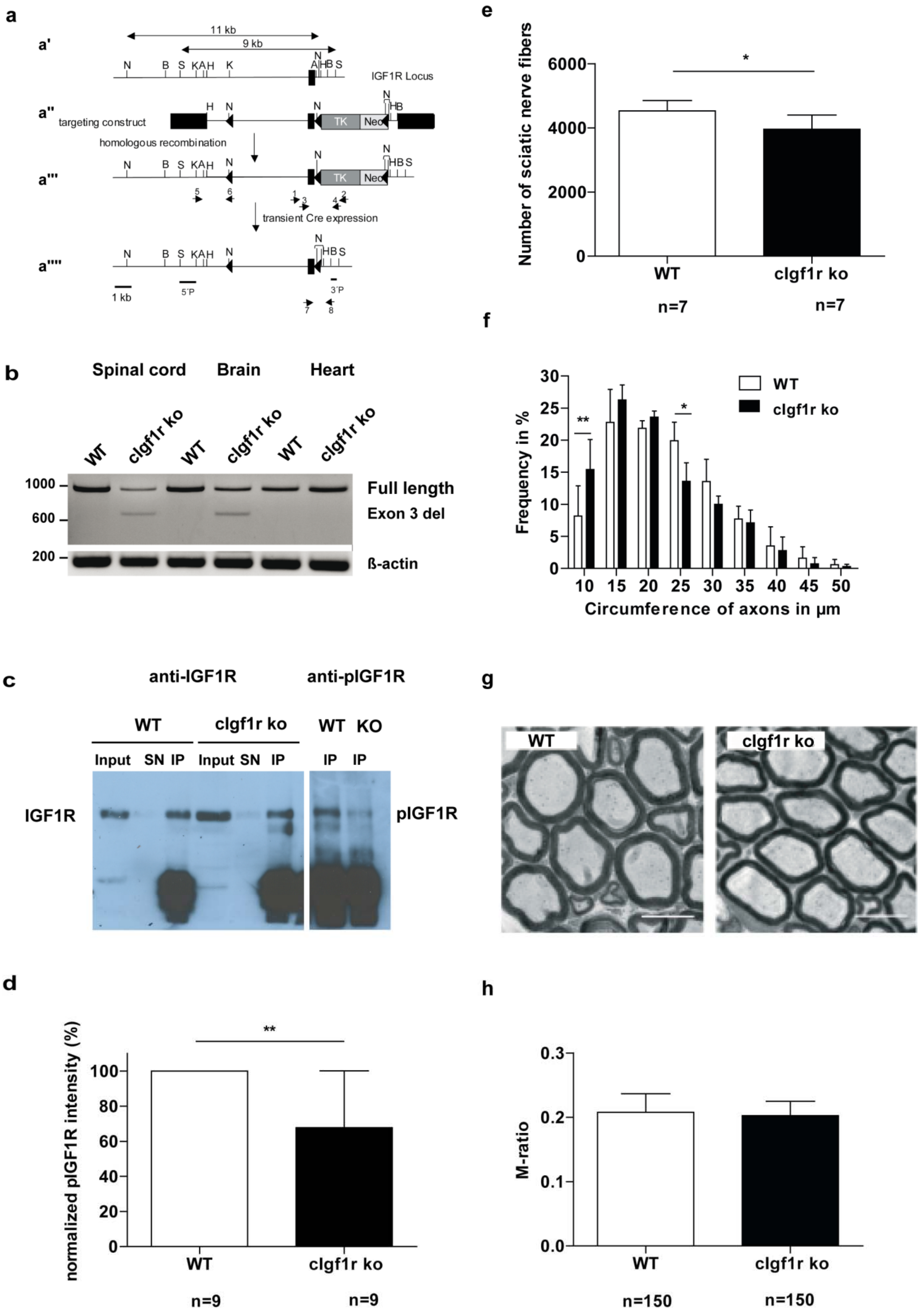

a
a'
11 kb
9 kb
IGF1R Locus
a''
targeting construct
homologous recombination
TK
Neo
a'''
transient Cre expression
a''''
1 kb
5'P
3'P
b
Spinal cord
Brain
Heart
WT
cIgf1r ko
1000
600
200
Full length
Exon 3 del
ß-actin
c
anti-IGF1R
anti-pIGF1R
WT
cIgf1r ko
WT KO
Input SN IP Input SN IP
IP IP
IGF1R
pIGF1R
d
normalized pIGF1R intensity (%)
WT
cIgf1r ko
n=9
n=9
e
Number of sciatic nerve fibers
WT
cIgf1r ko
n=7
n=7
f
Frequency in %
WT
cIgf1r ko
Circumference of axons in µm
g
WT
cIgf1r ko
h
M-ratio
WT
cIgf1r ko
n=150
n=150

Fig. A4 *cIgf1r ko* mice show axon and motoneuron degeneration similar to *Igfbp5 tg+* mice

a Map of the mouse genomic *Igfr* locus. (a') Exon III is represented by the shaded box, (a'') targeting construct, (a''') homologous recombinant and (a'''') floxed *Igfr* exon III locus N: *Nhe*I, B: *Bam*HI, S: *Sac*I; K: *Kpn*I, A: *Avr*II, H: *Hind*III, t loxP sites, 5´P and 3´P: external probes for 5´and 3´ends respectively. Primers: 1: TKPROM2, 2: BAMP3, 3: TKPROM3, 4: BAMP4, 5: EXTLOXP, 6: loxP2, 7: X3SEQ2, 8: RECSEQ3. **b** Reverse transcriptase PCR revealed exon 3 deletion in neuronal tissue of *cIgf1r ko* mice. **c d** Immunoprecipitation of the IGF1 receptor from spinal cord extracts of 4-day-old mice and subsequent analysis of its phosphorylation showed decreased activation levels in *cIgf1r ko* animals. SN = supernatant, IP = immunoprecipitation, **e** 6-month-old *cIgf1r ko* mice showed axon loss in the sciatic nerve. **f** The frequency of fibers with a circumference between 20–25 µm was decreased by 6 % in the sciatic nerve of 6-month-old *cIgf1r ko* mice. **g** Representative photomicrographs of myelinated fibers in semithin cross-sections of the sciatic nerve of 6-month-old wild-type and *cIgf1r ko* animals. Scale bar 10 µm. **h** The M-ratio was unchanged in 6-month-old *cIgf1r ko* mice. All results are presented as mean ± SD determined from the analysis of *n* mice per genotype (*$p < 0.05$, **$p < 0.01$, and ***$p < 0.001$). The white bars represent wild-type, and the black bars represent *cIgf1r ko* mice.

Fig. A5

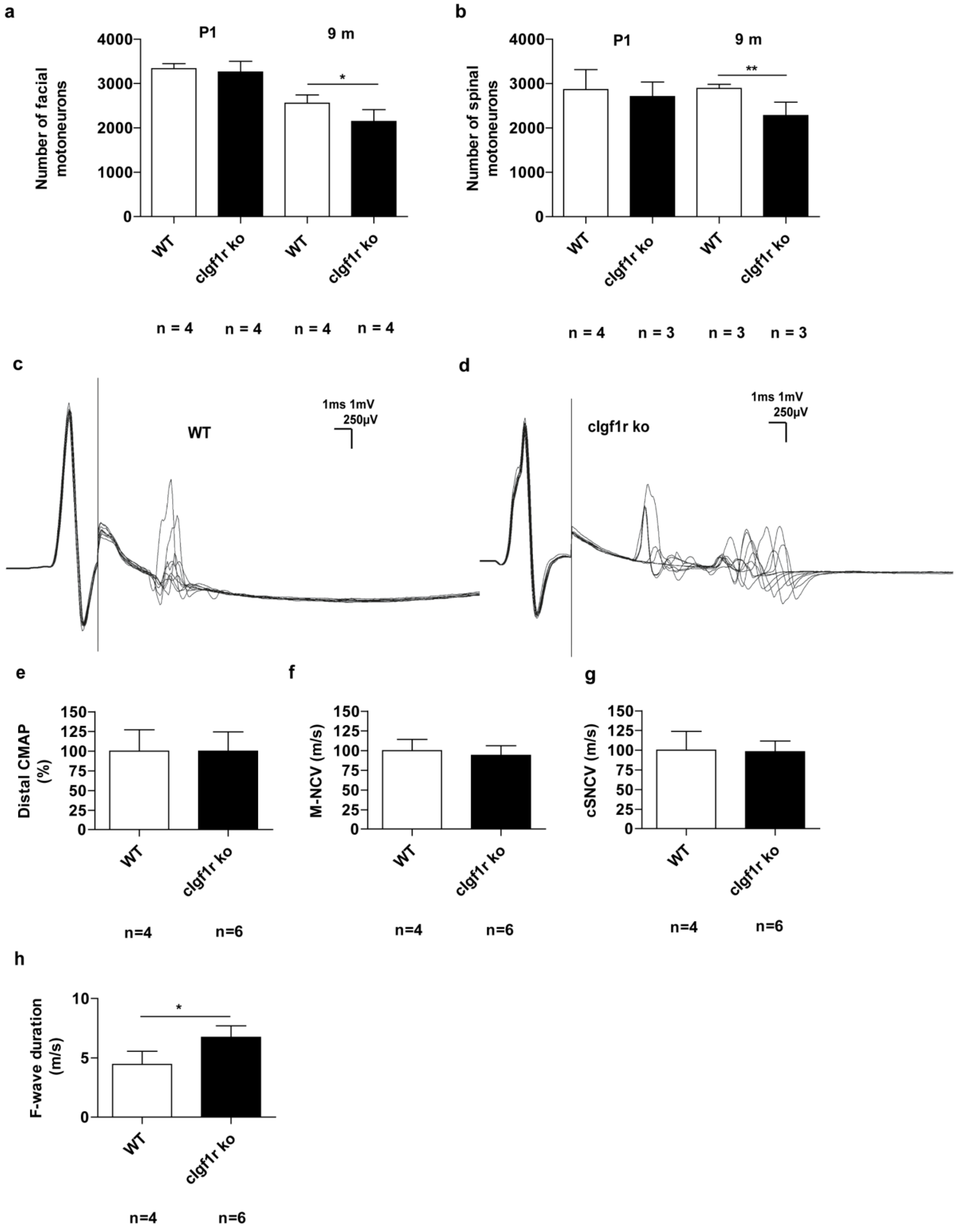
a
b
P1
9 m
Number of facial motoneurons
Number of spinal motoneurons
WT
clgf1r ko
n = 4
n = 3
c
d
1ms 1mV
250µV
clgf1r ko
e
f
g
h
Distal CMAP (%)
M-NCV (m/s)
cSNCV (m/s)
F-wave duration (m/s)
n=4
n=6

Fig. A5 *cIgf1r ko* mice show motoneuron and axon loss

a b In 9-month-old *cIgf1r ko* mice, the number of facial nucleus motoneurons was reduced by 16 %, and the number of lumbar spinal motoneurons by 21 %. **c d** Nerve conduction velocity studies in *cIgf1r ko* mice and wild-type littermates. **e** Distal CMAP amplitudes were not altered in *cIgf1r ko* mice compared to controls. **f** Motor nerve conduction velocities (M-NCV) were not significantly altered. **g** Compound sensory-motor nerve conduction velocities (cSNCV) were not significantly altered. **h** F-wave duration was increased in *cIgf1r ko* mice. All results are presented as mean ± SD determined from the analysis of *n* axons/mice per genotype (*$p < 0.05$, **$p < 0.01$, and ***$p < 0.001$). The white bars represent wild-type, and the black bars represent *cIgf1r ko* mice.

Supplementary Tables

Table A1 Microarray expression analysis of sural nerve biopsies from a control and a DNP patient: Relative expression of transcripts related to IGF signaling.

Upregulated	GENE SYMBOL	NP-Control Fold change
insulin-like growth factor binding protein 5	IGFBP5	7.48
insulin-like growth factor 1 receptor	IGF1R	5.28
insulin-like growth factor binding protein 7	IGFBP7	4.59
insulin-like growth factor 2 receptor	IGF2R	2.87
insulin-like growth factor 2 mRNA binding protein 3	IGF2BP3	1.53
insulin-like growth factor binding protein-like 1	IGFBPL1	1.35
insulin-like growth factor binding protein 2, 36kDa	IGFBP2	1.27
IGF-like family member 2	IGFL2	1.26
**Downregulated**	**GENE SYMBOL**	**NP-Control Fold change**
insulin-like growth factor 1 (somatomedin C)	IGF1	4.65
insulin-like growth factor 2 mRNA binding protein 1	IGF2BP1	2.26
insulin-like growth factor 2 mRNA binding protein 2	IGF2BP2	2.19
insulin-like growth factor binding protein 4	IGFBP4	2.07
insulin-like growth factor binding protein, acid labile subunit	IGFALS	1.98
IGF-like family member 1	IGFL1	1.74
insulin-like growth factor binding protein 3	IGFBP3	1.37
insulin-like growth factor binding protein 6	IGFBP6	1.27
insulin-like growth factor binding protein 1	IGFBP1	1.10

Table A2 Clinical data of patients used for Western blot analysis in Fig.1a, b.

	DNP	DNP and CIDP	CIDP	Disease controls	Healthy controls
N	6	4	9	3	5
Median age (range) [years]	60.5 (47-88)	68 (57-73)	69 (49-74)	64 (54-67)	33 (31-65)
Gender M; F	2; 4	1; 3	5; 4	1;2	4;1
Diabetes Type I:II	1:5	0:4	n.a.	n.a.	n.a.
Median disease duration until biopsy (range) [years]	1.4 (0.01-9)	1 (1-2)	2 (0.1-9)	2 (0.5-5)	n.a.
Clinical type and severity of neuropathy	SM: 6<br>Mild: 1<br>Moderate: 4<br>Severe: 1	SM: 4<br>Mild: 1<br>Moderate: 3<br>Severe: 0	SM: 7; M: 2<br>Mild: 1<br>Moderate: 5<br>Severe: 3	SM: 3<br>Mild: 0<br>Moderate: 3<br>Severe: 0	n.a.
Electrophysiological type of neuropathy	A: 6	A: 2<br>D: 2	D: 9	A: 3	n.a.
Sural nerve pathology	A: 6	D+I: 3	D: 2<br>A/D +I: 2<br>D+I: 5	A: 3	n.a.

Demographic and clinical data of all patients investigated in this study. DNP: Diabetic neuropathy. CIDP: Chronic inflammatory demyelinating neuroradiculopathy. Disease controls: One case of amyotrophic lateral sclerosis, spinal muscular atrophy, and vitamin B12 deficiency each. n.a.: not applicable. Type of neuropathy: M = pure or predominantly motor; SM = sensorimotor; A = predominantly axonal; D = predominantly demyelinating; A/D = both axonal and demyelinating; I = inflammatory

Table A3 Morphometric analysis of control and *Bp5 tg* + mice

Tissue	Age	Control mean ±SD	(n)	*Bp5 tg+*	(n)	Ratio *Bp5 tg+*/ Control	P-value
Facial motoneuron number	P1	3312±53	3	3258±231	3	0.984	0.717
	P21	3404±212	4	3015±264	4	0.886	0.244
	5-6 months	3260±182	6	2696±266	7	0.827	0.002
Lumbar spinal motoneurons	4 months	3066±194	3	2758±170	3	0.900	0.108
	5-6 months	2951±100	4	2373±140	6	0.804	0.0001
	16 months	2735±101	3	2207±251	3	0.807	0.0281
Phrenic nerve fiber number	P21	298±27	4	252±14	6	0.846	0.008
	5-6 months	274±13	5	247±12	6	0.901	0.049
	16 months	236±6	3	211±4	3	0.894	0.0036
Sciatic nerve fiber number	5-6 months	4628±172	4	3968±443	6	0.858	0.023

Table A4 Morphometric analysis of control and *cIgf1r ko* mice

Tissue	Age	Control mean ±SD	(n)	*cIgf1r ko*	(n)	Ratio *cIgf1r ko/* Control	P-value
Facial motoneuron number	P1	3337±115	4	3262±242	3	0.978	0.598
	9 months	2558±186	4	2147±267	4	0.839	0.045
Lumbar spinal motoneurons	P1	2866±451	4	2710±325	3	0.946	0.634
	9 months	2890±96	4	2281±300	4	0.789	0.008
	16 months	2718±88	3	2049±260	3	0.754	0.014
Phrenic nerve fiber number	6 months	246±19	4	219±4	4	0.890	0.034
	16 months	239±6	3	218±7	3	0.912	0.024
Sciatic nerve fiber number	6-7 months	4527±328	7	3956±445	7	0.874	0.018

Supplementary Methods

Microarray analysis

RNA for microarray was extracted using TRIzol (Invitrogen, Life Technologies). RNA integrity was assayed and comparted using a BioAnalyzer (Agilent, Santa Clara, CA). Only RNA samples with RNA integrity numbers (RIN) higher than 6 were used for subsequent microarray analysis. Of the RNA samples isolated, only one from a patient with diabetic neuropathy and one from healthy control fulfilled the criterion of an integrity number higher than 6 for the isolated RNA, and therefore these samples were used for microarray analysis. Total RNA (100 ng) was labeled (IVT-express kit, Affymetrix, USA). Labeled cRNA was fragmented for 25 minutes and hybridized to the human Genome U133 Plus 2.0 GeneChip® (Affymetrix). Data analysis was performed using different R packages from the Bioconductor project (www.bioconductor.org). Resulting signal intensities were normalized by quantile normalization [1]. Data sets were comparted by density plot and RNA degradation plot. For functional clustering, the Database for Annotation, Visualization and Integrated Discovery (http://david.abcc.ncifcrf.gov/home.jsp) has been used.

Generation of *Igfbp5* transgenic and motoneuron-specific *Igf1r* knockout mice (*cIgf1r ko*)

Isolation of *Igfbp5* cDNA clones

A λ-ZAPII mouse brain cDNA library was screened using an 873 bp PCR - generated cDNA probe (forward primer IGFBP5-1 5'-GCC CCG AGG TAA AGC CAG ACT -3'; reverse primer IGFBP5-3 5'-GGA TAG GGG GAG GAA GGG AGG -3') representing the entire coding sequence and 48 bp of 5'untranslated region. Clones with the full-length cDNA were

plaque purified and high titer stocks prepared. Excision of the integrated pBS II SK vectors containing cDNA inserts in the polylinker EcoRI site was carried out using the EXASSIST/SOLR system (Stratagene, La Jolla, Ca) according to the manufacturer's instructions and clone identity was confirmed by DNA sequencing.

Construction of a NF-L-*Igfbp5* DNA targeting vector

A 1.8kb *Eco*RI fragment containing the *Igfbp5* cDNA (excised from pBSII SK using the polylinker *Eco* RV site and an NseI site in the 3′ UTR) and the polyA region from pMC-Cre (S1) were isolated and blunt-end ligated into pKS.NFL (gift from J. P. Julien) between blunted XhoI/ClaI sites. The 8 kb fragment including the human *NF-L* promoter, mouse *Igfbp5* cDNA, the polyA signal from pMC-Cre and exons 2-4 of the *NF-L* gene (downstream region) was excised from NFL-BP5 using *Sma*I. The fragment was gel purified twice and purified with Nucleotrap resin (Macherey and Nagel, Düren, Germany), extracted with butanol, then desalted and further purified over a Schleicher and Schuell (Dassel, Germany) Elutip. Eluted DNA was extracted with phenol/chloroform and chloroform and then precipitated with ethanol. DNA for microinjection was resuspended in sterile-filtered injection buffer (5 mM Tris-HCl, pH 7.4, 0.1 mM EDTA) at approximately 500 copies/pl. DNA and microinjected into fertilized mouse oocytes.

Identification and characterization of *Igfbp5* transgenic mouse lines

Nine founders were identified by PCR and Southern blot analysis of tail DNA. The PCR reaction used for genotyping was carried out with forward primer NFL-SEQ 5′- TCG CAG GCT GCG TCA GGA G -3′ and reverse primer BP5PCR 5′- CTT GCA GGT AGA GCA GGT GCT CTC -3′, 45 cycles of 45 " at 94 °C, 45 " at 53 °C, 30 " at 72 °C. For Southern blots, tail DNA was digested with *Xho*I, and probed with the radiolabeled *Igfbp5* PCR product described above.

Construction of the floxed *Igf1r* targeting vector

A 600 bp *BamH*I/*Hind*III intron III fragment was subcloned between the *Bam*HI and *Hind*III sites of pBSII (Stratagene) in which the *Kpn*I site had been destroyed by end-fill and religation. The resulting plasmid was digested with *Hind*III, and a 6 kb *Hind*III fragment containing part of intron II, exon III and the region of intron III extending to the *Hind*III site was inserted. Correct orientation was confirmed by sequencing. After linearization of this plasmid with *Kpn*I, a 100 bp fragment containing a single loxP site (isolated from pGH1/loxP which was derived from pGH1 by addition of another loxP site 5` to the neo cassette (S1) with *EcoR*I/*Pst*I) was inserted by blunt-end ligation. The positive-negative selection cassette (containing the neomycin resistance and the Herpes simplex virus thymidine kinase genes both under the control of thymidine kinase promoters flanked by loxP sites in parallel orientation) was isolated from pGH1/loxP by excision with *Xba*I/*Nsi*1. The plasmid backbone was simultaneously digested with *Sca*I to enable gel purification of the 3 kb selection cassette. Finally, the single loxP targeting construct intermediate was digested with *Avr*II and the selection cassette flanked by loxP sites was inserted by blunt end ligation to generate the complete targeting vector. Parallel orientation of all three loxP sites was confirmed by sequencing.

Isolation of homologous recombinant floxed *Igf1r* ES cell clones.

The targeting vector (25 µg) was linearized by digestion with *Cla*I and electroporated into R1 embryonic stem cells that were selected in G418 (400 mg/ml). The G418-resistant ES colonies were picked and subjected to nested PCR analysis using primers TKPROM2 (5′-AAC CAC ACT GCT CGA CAT TGG -3′) and BAMP3 (5′- GAA TTG TTA CGA ATA CTG GAG ACT GG -3′) for the first round and TKPROM3 (5′- GCA AAA CCA CAC TGC TGC ACC -3′) and BAMP4 (5′- GAT CTA GGA CCT TCT ACA AGG TGG G -3′) for the second round using 5 µl of the first round PCR. Positive single clones were screened for

correct integration at the 5′- end using the primers EXTLOXP 5′- CTT CCC ACG TTG GAC TCG GAA GC -3′, and loxP2 5′- GGT CTG AAG AGG AGT TTA CGT CC -3′. The PCR product (1.6 kb) extended from a region of intron 2 of the *Igf1r* gene lying outside the floxed *Igf1r* construct to the 5′loxP site in intron 2. ES cell clones positive for both PCR assays were subjected to Southern analysis. DNA was digested with either *Sac*I (and hybridized with a radiolabeled 330 bp *Dra*I / *BamH*I 3′-external probe) or with *Nhe*I followed by hybridization with 5′external probe of 1.3 kb corresponding to the *Kpn*I/*Hind*III fragment immediately upstream of the targeting construct. Southern blot filters were stripped and reprobed with a Neo cDNA probe and homologous recombinant clones with no additional random integration events were selected for transfection with pMC-Cre [5]. For each clone 2 x 10^6 cells were transiently transfected with pMC-Cre DNA (6 µg) and plated onto primary mouse embryonic fibroblast feeder layers in 10cm culture dishes. After 3 days, cells were split 1:4 and negative selection with ganciclovir (2 µM) was initiated. After a further 5 days ganciclovir was removed and colonies were allowed to grow without selection for 2-3 days before picking. DNA was prepared from single colonies and screened by PCR across the former position of the selection cassette with the following primers: X3SEQ2 5′- CAG GAG TGT CCC TCA GGC TTC ATC -3′. To confirm Cre-mediated deletions of floxed alleles by Southern blot ES cell genomic DNA was digested with *Hind*III and probed with an internal 1.6 kb *Kpn*I/*Hind*III fragment to detect Type I and Type II deletions [4] in addition to the homologous recombinant allele. The Southern blot was reprobed with the Neo cDNA probe to confirm the deletion of the selection cassette and the absence of random integration events. Chimeric mice were produced by microinjection of floxed *Igf1r* clones. Chimeric males were mated with C57BL/6 females and germline transmission of the floxed allele was verified by Southern blot analysis of tail DNA from agouti colored F1 offspring. For neuron-specific inactivation of *Igf1r*, mice homozygous for the floxed *Igf1r* allele ($Igf1r^{loxP/loxP}$) were crossbred with a mouse line that expresses Cre recombinase under control of the human neurofilament light chain

(NF-L) promoter and exhibits Cre expression in specific populations of neurons, including facial and spinal motoneurons [11]. Neuron-specific *Igf1r* knock-out animals (*cIgf1r ko)* had the genotype NF-L-Cre, *Igf1r*$^{loxP/loxP}$. Control animals were either *Igf1r*$^{loxP/loxP}$ or *Igf1r*$^{WT/WT}$ (referred to hereafter as wild-type).

Reverse transcription (RT)-PCR and quantitative real-time RT-PCR (qRT-PCR)

For quantitative real-time RT-PCR (qRT-PCR), total RNA was converted into cDNA using the LightCycler Fast start DNA Master SYBR Green I kit (Roche). Sequences of primers used for qRT-PCR were: *Igfbp5* (sense, 5'- CAAGAGAAAGCAGTGTAAGCC -3'; *Igfbp5* antisense, 5'- CACTCAACGTTACTGCTGTC -3'); β-Actin (sense, 5'- GCCAACCGTGAAAAGATGAC -3'; antisense, 5'- GGCGTGAGGGAGAGCATAG -3'). Thermal cycling was performed on a Light Cycler 1.5 (Roche). *Igfbp5* mRNA levels were calculated using $2^{-\Delta\Delta Ct}$ method [8]. To ascertain the level of exon 3 deletion in *cIgf1r ko* mice in the spinal cord, RT-PCR with following exon 3 spanning primer set was performed (sense primer GGATGCGGTGTCCAATAACT, antisense primer CTCCGTTGTTCCTGGTGTTT, full length product size: 971 base pairs, product with exon 3 deletion: 658 base pairs).

Substance P ELISA

To assess Substance P in footpads, the Enzyme Immunoassay Kit (Cayman Chemical Co., Ann Arbor, MI, USA) was used. Footpads were homogenized in 0.05 M acetic acid buffer including 1 μg/mL pepstatin, 2 μg/mL aprotinin and 5 μg/mL leupeptine, then boiled for 10 min. After the homogenates were centrifuged at 14000 rpm for 30 min, the supernatant was dried under vacuum using a SpeedVac apparatus (Savant, Farmingdale, NY) and dissolved in EIA buffer. The assays were processed in duplicates according to the manufacturer's instructions. Absorbance was determined by Thermo Scientific microplate reader (405 nm)

and converted to SP content level in EIA buffer (pg/mL) by normalizing to the standard curve. For statistical analysis 6 measured values from 3 independent wild-type and *Igfbp5* transgenic mice were taken into account.

Quantitative morphometry on cross sections of the Igfbp5 tg+ saphenous nerve

Ten to eleven–month-old *Igfbp5 tg+* and wild-type mice were killed under anesthesia and saphenous nerves were dissected and immersion-fixed with 4 % paraformaldehyde in 0.1 M phosphate buffer (wt: n = 7; *Bp5* tg+: n = 6). Saphenous nerves were processed as mentioned for the phrenic and sciatic nerve. Cross sections were photographed using a Olympus FV 1000 confocal microscope in transmission scan modus. Light micrographs of entire nerve bundles were analyzed for axon perimeter and g-ratio by an observer blinded to the genotype. One hundred randomly selected fibers were analyzed per animal. We implemented a plug-in for the ImageJ software (http://rsbweb.nih.gov/ij), which allowed for semiautomated analysis of randomly selected sets of fibers. Plug-in and source code are available online (http://gratio.efil.de/) of myelinated axons were determined on a representative image of semithin sections (100×). To determine the size distribution of myelinated axons in saphenous nerve cross sections, perimeters of 100 randomly chosen fibers per mouse in a representative picture of semithin sections (representing 19–29 % of each complete nerve cross section) were measured.

Quantification of motoneurons in the facial nucleus and lumbar spinal cord

We determined the number of motoneuron cell bodies in the facial nucleus of newborn, 3-week and 5- to 6-month-old and in the lumbar spinal cord of 5- to 6-month-old *Igfbp5 tg+* and wild-type mice derived from mouse lines 8 and 9 as described previously [9]. In addition, the number of facial and spinal cord motoneurons was counted in newborn and 6- to 8-month-old *cIgf1r ko* and wild-type mice. Animals were transcardially perfused with 4 %

paraformaldehyde in phosphate buffered saline at pH 7.4 and the brainstem and lumbar spinal cord (L1-L6) were dissected. Serial sections were cut from the brain stem region (7 µm) including the facial nuclei and lumbar spinal cord (15 µm). After Nissl staining, motoneurons were counted in every 5 th (facial nucleus) or 10 th section (spinal cord) and the raw counts were corrected for split nuclei [9].

Thermal and mechanical nociception assays

All behavioral assays were conducted on 10- to 11-month-old *Igfbp5 tg+* and wild-type mice (wild-type, n = 14, transgenic, n = 18). Experiments were done during the light cycle. Animals were acclimated for 20 minutes to their testing environment prior to all behavioral assays that were done at room temperature (~22 °C). Experimenters were blind to the genotype of the mice during testing. Paw withdrawal latencies to heat were measured according to the method of Hargreaves [6] applying a standard Ugo Basile Algesiometer (Comerio, Italy). The heating rate of the surface during the paw Hargreaves test was 1.6 °C per second which is appropriate for stimulating C-fibers. The animals were placed on a glass surface and a radiant heat source was positioned under one hind paw. The time to paw withdrawal was recorded automatically. To avoid tissue damage the time limit for heat application was set to 15 seconds. Each hind paw was tested three times. The von-Frey test based on the up- and- down-method was used to test for the paw withdrawal thresholds to tactile stimulation [3]. Animals were placed in plexiglas cages on a wire mesh. The plantar surface of the hind paws was touched with a von Frey monofilament starting at a hair value of 0.69 g. When the animal withdrew its hind paw upon administration of mild pressure the next finer von Frey filament was used. If the animal did not react to this stimulation, the next stronger von Frey filament was applied. The range of von Frey monofilament strength used was 0.07-1.20 g. Each hind paw was tested six times consecutively. The 50 % withdrawal

threshold (i.e. force of the von Frey hair to which an animal reacts in 50 % of the administrations) was recorded.

Forelimb grip strength test

The grip strength test was performed with a grip strength meter (Chatillon). 2- month and 9-month old *Igfbp5* transgenic and wild-type mice were allowed to grasp a triangular ring attached to a force transducer and gently pulled away by the base of the tail until the grip was broken. The test was repeated five times and the mean peak force values (g) were calculated for each animal.

Accelerating rotarod

Motor coordination and balance were assessed on a rotarod device (Ugo Basile) by placing 2-month and 9- month old *Igfbp5* transgenic and wild-type mice on a rotating drum and measuring the time for which each animal was able to maintain its balance on the rod as latency time to fall (seconds). After an initial phase at 18 rpm, speed of the rotarod was accelerated within 2 seconds to 30 rpm, and mice tested for another 70 sec period. Mice that fell were re-tested for a total of three consecutive trials. All trials were used for statistical analysis.

Electrophysiological analysis

Motor nerve conduction testing: First experiments were done on a Toennies electromyograph and motor nerve conduction velocity (motor NCV) was measured under general anesthesia with HypnormR (Janssen, Beerse, Belgium) in the sciatic nerve as described [2]. In the later experiments with conditional mutants the studies were done on a digital Neurosoft-Evidence 3102 electromyograph (Schreiber & Tholen Medizintechnik, Stade, Germany). Here, mice

were anesthetized by intraperitoneal injection of ketamine (Pfizer) and xylazine (Bayer) in saline with 100 μg per gram bodyweight and 7 μg per gram, respectively, which does not reduce F-waves persistence [10]. Proximal and distal compound motor action potential (CMAP) amplitudes, proximal and distal F latencies (F Lat.), proximal and distal motor latencies (M.Lat.) were determined according to a standard protocol as described [7, 12]. After supramaximal stimulation of the tibial and peroneal nerve at the ankle (distal) and stimulation of the sciatic nerve at the sciatic notch (proximal), the compound muscle action potentials (CMAP) were recorded with steel needle electrodes in the foot muscles and CMAP amplitudes (millivolts) and latencies (milliseconds) were measured and nerve conduction velocities calculated (meters per second). Compound sensory - motor nerve action potentials (cSNAP) were measured by stimulation at the ankle and recording from the sciatic notch, and nerve conduction velocities were calculated. F-waves were recorded from the foot muscles after 10 repetitive supramaximal stimuli at the ankle at stimulation rates of 0.3, 1, and 10 Hz and F-waves persistence (in percent) and latencies were calculated [12]. Typically, H-reflexes if present were superimposed on F-waves and were reduced with higher stimulation frequencies which allowed for discerning and assessing isolated F-waves [10].

References for Supplement:

1 Bolstad BM, Irizarry RA, Astrand M, Speed TP (2003) A comparison of normalization methods for high density oligonucleotide array data based on variance and bias. Bioinformatics 19: 185-193

2 Bremer J, Baumann F, Tiberi C, Wessig C, Fischer H, Schwarz P, Steele AD, Toyka KV, Nave KA, Weis Jet al (2010) Axonal prion protein is required for peripheral myelin maintenance. Nature neuroscience 13: 310-318 Doi 10.1038/nn.2483

3 Chaplan SR, Bach FW, Pogrel JW, Chung JM, Yaksh TL (1994) Quantitative assessment of tactile allodynia in the rat paw. Journal of neuroscience methods 53: 55-63

4 Gu H, Marth JD, Orban PC, Mossmann H, Rajewsky K (1994) Deletion of a DNA polymerase beta gene segment in T cells using cell type-specific gene targeting. Science 265: 103-106

5 Gu H, Zou YR, Rajewsky K (1993) Independent control of immunoglobulin switch recombination at individual switch regions evidenced through Cre-loxP-mediated gene targeting. Cell 73: 1155-1164

6 Hargreaves K, Dubner R, Brown F, Flores C, Joris J (1988) A new and sensitive method for measuring thermal nociception in cutaneous hyperalgesia. Pain 32: 77-88

7 Krieger F, Elflein N, Saenger S, Wirthgen E, Rak K, Frantz S, Hoeflich A, Toyka KV, Metzger F, Jablonka S (2014) Polyethylene glycol-coupled IGF1 delays motor function defects in a mouse model of spinal muscular atrophy with respiratory distress type 1. Brain : a journal of neurology 137: 1374-1393 Doi 10.1093/brain/awu059

8 Livak KJ, Schmittgen TD (2001) Analysis of relative gene expression data using real-time quantitative PCR and the 2(-Delta Delta C(T)) Method. Methods 25: 402-408 Doi 10.1006/meth.2001.1262

9 Masu Y, Wolf E, Holtmann B, Sendtner M, Brem G, Thoenen H (1993) Disruption of the CNTF gene results in motor neuron degeneration. Nature 365: 27-32 Doi 10.1038/365027a0

10 Nowicki M, Baum P, Kosacka J, Stockinger M, Kloting N, Bluher M, Bechmann I, Toyka KV (2014) Effects of isoflurane anesthesia on F-waves in the sciatic nerve of the adult rat. Muscle & nerve 50: 257-261 Doi 10.1002/mus.24150

11 Schweizer U, Gunnersen J, Karch C, Wiese S, Holtmann B, Takeda K, Akira S, Sendtner M (2002) Conditional gene ablation of Stat3 reveals differential signaling requirements for survival of motoneurons during development and after nerve injury in the adult. The Journal of cell biology 156: 287-297 Doi 10.1083/jcb.200107009

12 Zielasek J, Martini R, Toyka KV (1996) Functional abnormalities in P0-deficient mice resemble human hereditary neuropathies linked to P0 gene mutations. Muscle & nerve 19: 946-952 Doi 10.1002/(SICI)1097-4598(199608)19:8<946::AID-MUS2>3.0.CO;2-8
